# The First Application of ^1^H NMR Spectroscopy for the Assessment of the Authenticity of Perfumes

**DOI:** 10.3390/molecules26113098

**Published:** 2021-05-22

**Authors:** Barbara Pacholczyk-Sienicka, Grzegorz Ciepielowski, Łukasz Albrecht

**Affiliations:** Institute of Organic Chemistry, Faculty of Chemistry, Lodz University of Technology, Zeromskiego 116, 90-924 Lodz, Poland; grzegorz.ciepielowski@p.lodz.pl

**Keywords:** authentication of perfumes, flavors, nuclear magnetic resonance, counterfeits

## Abstract

The manufacture of counterfeit goods is one of the world’s largest underground businesses and is rapidly growing. Counterfeits can lead not only to the loss of profit for honest producers but also have a negative impact on consumers who pay excessive prices for poor quality goods that may result in health or safety problems. The perfume industry is constantly vulnerable to counterfeits, particularly in the fast developing market of “smell-alike” designer-inspired perfumes because these prompt the identification of the methods that classify their quality. In this paper, the application of proton nuclear magnetic resonance (^1^H NMR) spectroscopy is employed for the first time to authenticate perfumery products. The molecular composition of several types of authentic brand fragrances for women was compared with cheap inspired equivalents and fakes. Our approach offers the prospect of a fast and simple method for detecting counterfeit perfumes using ^1^H NMR spectroscopy.

## 1. Introduction

The globalization of products with recognized market position such as beverages, medicines, food and perfumes has led to the expansion of counterfeiting. These fake goods purchased through Internet shops and websites may cost less than the originals, but they are of inferior quality [1,2]. Moreover, these counterfeit goods may be dangerous because they do not undergo the same rigorous safety testing conducted by honest manufacturers. These inferior imitations affect not only companies by damaging the brand’s reputation but also consumers, who may be unaware that they do not comply with safety standards and could be dangerous. For the most part, these products do not undergo dermatological testing and may contain chemicals that can cause skin reactions such as contact allergies or dermatitis [3,4].

Nowadays, fragrances are found in almost all cosmetics and personal care products (e.g., perfumes, deodorants, aftershave and skin lotions), hair products, facial cleansers and sunscreen as well as cleaning and laundry products. However, a given fragrance is typically a mixture of several dozen to several hundred chemicals that may cause the allergic contact dermatitis [5,6,7,8,9]. The last data indicated that more than 3000 chemical compounds are used in fragrance mixtures but only 82 substances (54 single chemicals, 28 natural extracts) were classified as contact allergens in humans by The Scientific Committee on Consumer Safety [2]. A perfume usually contains 30 to 50 (sometimes even 200) ingredients responsible for its unique fragrance, which come from nature or chemical synthesis. The composition and scent of the essential oils depend not only on the plant’s origin, conditions of collection, storage, transport and drying but also on the process of oil production. It is particularly important to monitor contaminants that may be present in oils obtained from plants as they may contain traces of toxic or carcinogenic pesticides [7,8]. Due to all of the properties of essential oils, the high-quality natural oils are very expensive. Their replacement by synthetic chemical compounds reduces the cost of perfume production, which mainly depends on the quality of oil used [6]. Owing to the high price of essential oils, manufacturers are adulterating their products while maintaining high prices. Two techniques of adulteration that are frequently used involve adding cheaper materials or dilution with water or other solvents.

The most commonly counterfeited scents are branded perfumes and toilet waters, but the imitations do not have the same quality or scent as the authentic ones [2,3]. Customers at first glance are not able to recognize a counterfeit because its external features strongly resemble those of the original even though it does not have the same molecular composition or properties. However, in some situations consumers can avoid buying a counterfeit simply by staying focused. Counterfeiters often use misspellings on the packaging to avoid liability for trademark theft (e.g. Boos instead of *Boss* or J’ader instead of *J’adore*).

Considering that perfumes are complex mixtures of various compounds, the determination of their ingredients is a difficult task. Several methods have been suggested for analysing fragrances such as chromatography [10,11,12], spectroscopy [13,14,15,16], mass spectrometry [17,18], and electronic nose [19,20]. Due to the fact that most perfume components are volatile or semi-volatile, gas chromatography coupled with mass spectrometry (GC–MS) is the technique of choice for qualitative and quantitative analyses of perfume ingredients. In recent years, GC–MS has been successfully employed in forensic reconstructions of crime scenes, such as identifying traces of volatiles on the clothes of a sexual assault victim because the fragrance may have been transferred between the victim and the attacker [21,22,23]. A technique that plays an important role in the fragrance industry is the artificial electronic olfactory system (EOS), especially concerning perfume counterfeiting, the quality control of raw and finished products, and the quantification of perfume concentration in an unknown sample. Spectroscopic techniques like Raman spectroscopy or NMR can be used for perfume analysis, the former having been applied to determine fragrance composition [24]. Interestingly, up to now NMR spectroscopy has only been applied to characterize the compositions of several perfumes and to separate the components of a given mixture based on diffusion coefficients using diffusion ordered spectroscopy (DOSY) [14,15,16].

Herein, we present a fast, simple and relatively inexpensive method to detect counterfeit perfumes. The goal of our study is to present the potential of NMR spectroscopy in the direct compositional analysis original perfumes and their counterfeits. We envision that it may be possible through the application of NMR spectroscopy with the aid of chemometric analysis. Within our studies, the ^1^H NMR spectra of original perfumes were compared with those of fakes and inspired equivalents. To the best of our knowledge, the use of ^1^H NMR spectra has never been reported for this purpose before. Our results offer the perspective for a fast, routine way to detect counterfeit perfumes by means of NMR spectroscopy.

## 2. Results and Discussion

**Figure 1:**
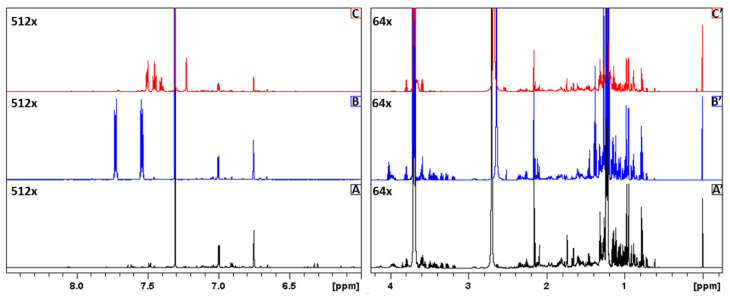
Representative ^1^H NMR spectra of authentic (**A**,**A′**), inspired (**B**,**B′**) and counterfeit (**C**,**C′**) samples in two spectral region from −0.25 to 4.25 ppm and from 6 to 8.5 ppm.

Representative ^1^H NMR spectra of the authentic, inspired and counterfeit perfumes are shown in the Figure 1. The main compounds characterizing perfume samples with their diagnostic ^1^H signals, chemical shifts δ_H_ and multiplicities are reported in Table S1 (see Supporting Information). Considering the usefulness of the applied approach to a direct analysis of perfume composition, we compared our results with data obtained by means of a GC–MS analysis for original *Light Blue* and its substitutes and counterfeit version [25]. Most of the identified compounds were also found in samples analyzed by NMR. Twenty-three molecules were identified on the basis of 1D and 2D NMR spectral analysis, while 34 compounds were detected by the GC–MS approach.

**Figure 2:**
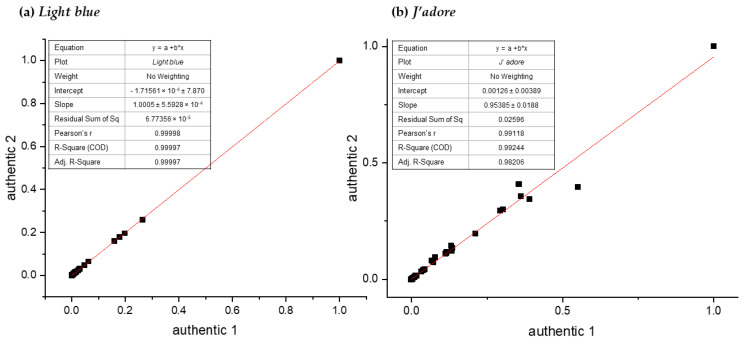
The comparison of the buckets from NMR spectra for two authentic samples of *Light blue* (**a**) and *J’adore* (**b**).

The main constraint of our study was the limited amount of authentic samples. So far, two authentic samples of *Light blue* and *J’adore* fragrances were obtained and tested. To evaluate chemical similarity, their spectra were calibrated with reference to the TMS signal, manually phased, and the baseline was corrected. Then by means of AMIX software spectra, they were segmented into buckets and normalized to the total sum of the integrals of all the buckets. After that, a linear regression analysis was conducted to compare the buckets for both spectra. For each comparison, the coefficient of determination (R^2^) of the fit was calculated. The results for *Light blue* are presented in Figure 2a, while those for *J’adore* are in Figure 2b. The highest value of R^2^ was obtained when two authentic samples of *Light blue* were compared (R^2^ = 0.99, Figure 2a) while, in the case of *J’adore* the coefficient of determination (R^2^) was 0.98. Given that a good correlation was obtained, further PCA analyses were conducted on one authentic sample for each of the fragrances tested. However, the slight differences observed for the two *J’adore* samples suggest that, for future applications of the developed method, several batches of authentic samples should be tested.

Due to the very large number of signals, the obtained NMR spectra were first analyzed by a main components analysis (PCA), which explains the variance structure of a set of variables through linear combinations of the principal components (PCs). The PCA scores discriminated the authentic perfume samples from the inspired and counterfeit samples based on selected proton signals detected by ^1^H NMR. For this purpose a full-spectrum PCA analysis was conducted as an input; however, the only observed difference in chemical composition referred to solvents. The outlying points belonged to ethyl alcohol, isopropyl alcohol, diethyl phthalate and cedrol. (PCA analysis for full spectrum bucket tables are shown in the Supplementary Information.) As a consequence, the full spectrum was divided into three spectral ranges of chemical shifts. The first covered the range from 0.6 to 3.0 ppm, the second from 3.0 to 6.0 ppm, and the last from 6.0 to 8.5 ppm. The interpretation of the results from the first interval did not allow the original samples to be distinguished from the inspired samples because all samples in this range of chemical shifts were very similar, so no statistically significant differences were observed.

**Figure 3:**
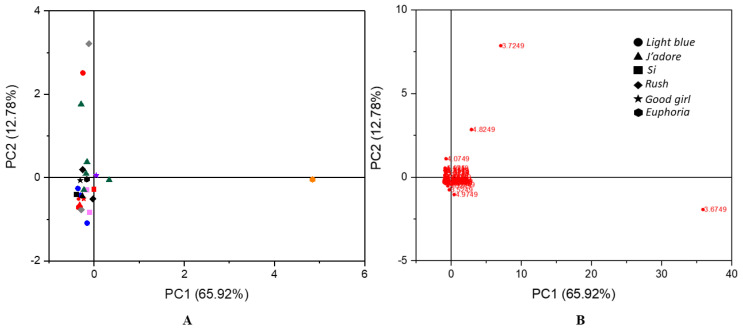
PCA scores plot (**A**) and the corresponding loading plot (**B**) generated from the ^1^H NMR spectra from the authentic perfume (black color, different shapes marked various scents) and counterfeit (red color, different shapes marked various scents) and inspired samples (colors, different shapes marked various scents) for the range of chemical shifts from 3.0 to 6.0 ppm.

Similar results were obtained for the range from 3.0 to 6.0 ppm. The respective clustering of PCA is shown in Figure 3A, and some outliers corresponding to the counterfeit sample of *Light blue* and the inspired samples of *Rush, J’adore*, and *Euphoria* were observed. This model described 78.7% of total variation in accordance with PC1 = 65.92% and PC2 = 12.78%. The loading plot of a principal component indicated buckets and therefore spectral regions that contributed significantly to it. As shown in Figure 3B, signals that were responsible for the similarity/dissimilarity between the observations corresponded to water (4.82 ppm), isopropanol (4.07) and isopropyl myristate (4.97 ppm), which were observed in non-original products. This was very characteristic of falsified and lower-quality products, because manufacturers often use more water or other solvents such as isopropyl myristate to reduce the cost of perfume production. Moreover, we observed that also ethyl alcohol was responsible for an outlying behaviour, which may indicate that poor quality ethanol was used for production of falsified and inspired fragrances. As a consequence, consumers buy poor quality goods at an excessive price. A PCA score plot based on the proton spectra of 26 samples is shown in the Figure 3.

**Figure 4:**
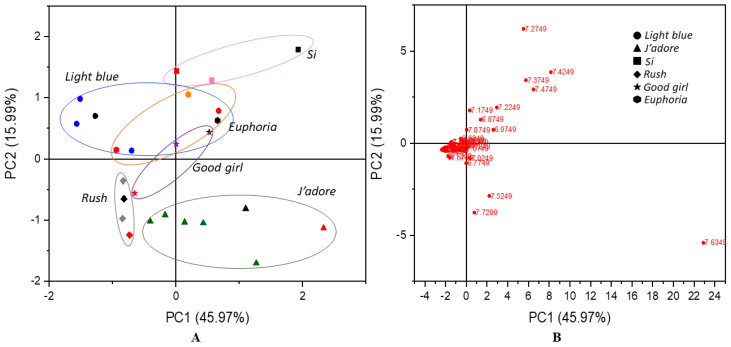
PCA scores plot (**A**) and the corresponding loading plot (**B**) generated from the ^1^H NMR spectra of the authentic perfume (black color, different shapes marked various scents) and counterfeit (red color, different shapes marked various scents) and inspired samples (colors, different shapes marked various scents) for the range of chemical shifts from 6.0 to 8.5 ppm.

As shown on the scores plot in Figure 3A, the authentic perfumes were not clearly separated from each other or from the inspired and counterfeit samples. Therefore, a PCA plot obtained for the range of chemical shifts from 6.0 to 8.5 ppm proved to be the most important because it allowed not only branded samples to be distinguished from non-original ones, but also enabled original fragrance compositions to be differentiated among each other. These results are shown in Figure 4. The total variation described by the scores of PC1 versus PC2 was 61.96%. This plot showed that the counterfeits differed a lot from the original perfumes and also occupied a different region in the plot compared to the inspired samples. This proved that the adulterated perfumes had different chemical compositions in the range of chemical shifts that corresponded to unsaturated compounds. It was particularly evident in the case of *Light Blue*, *Si*, *Euphoria* and *Good Girl*. Unlike counterfeit perfumes, these inspired samples showed considerable similarity with what was observed for authentic fragrances like *Rush*, *Light Blue*, *J’adore*, *Euphoria* and *Si*. The loading plot presented in Figure 4B indicates the signals that were associated to the similarity/dissimilarity between the observations of this study. The farthest outlying points belonged to diethyl phthalate (7.53 and 7.72 ppm), galaxolide (6.77 and 7.02 ppm), benzyl salicylate (6.88, 6.97, 7.27 and 7.87 ppm), 2-ethylhexyl *trans* 4-methoxycinnamate (overlapped with other signals 6.31 ppm and 7.63 ppm, characteristic coupling constant ^3^J = 16 Hz) and α-hexylcinnamaldehyde (7.17, 7.37, 7.42, and 7.47 ppm). Hexyl cinnamal (α-hexylcinnamaldehyde) is a widely used fragrance chemical because its scent resembles jasmine. In the perfume and cosmetics industry, synthetic hexyl cinnamal is used; however, it can be found naturally in chamomile oil. This ingredient may cause an allergic skin reaction and it is labelled by The European Chemicals Agency as a skin sensitizer. In turn, galaxolide is a synthetic aroma and known as one of the components in musk. Galaxolide is also used as a fixative that reduces the evaporation rate of the volatile components of perfumes. Benzyl salicylate acts as a preservative and fragrance in one. It gives products a sweet, floral aroma. Diethyl phthalate similar to isopropyl myristate is used as a solvent and fixative. 2-Ethylhexyl *trans*-4-methoxycinnamate is a cinnamate ester used as an ultraviolet light absorber to protect products.

**Figure 5:**
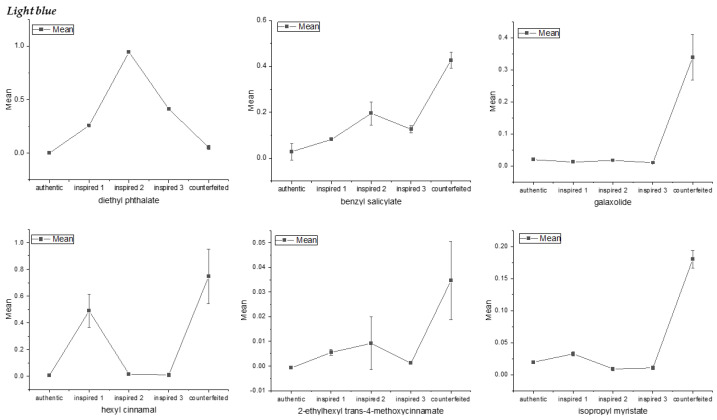
The comparison of the means integration values of ^1^H NMR signals for authentic, inspired and counterfeit *Light Blue* samples obtained by ANOVA. Note that five replications of each sample were conducted and the integration of five spectra was taken as an input for analysis.

**Figure 6:**
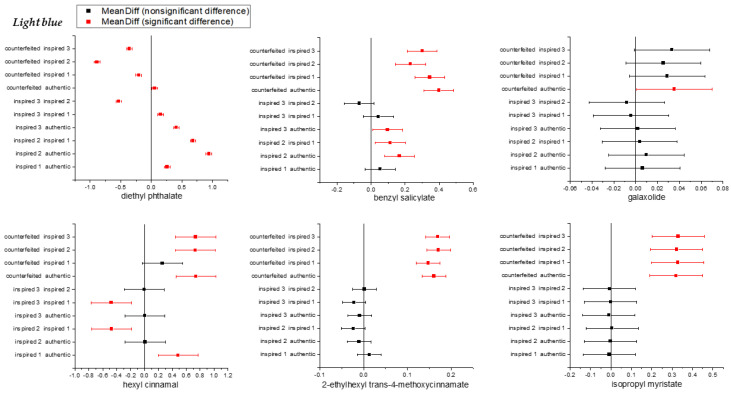
Means comparison plot from ANOVA (Tukey test, *p* < 0.05).

The significant differences in ^1^H NMR signals related to compounds between the authentic, inspired and counterfeit samples were determined using a one-way analysis of variance (ANOVA). It was performed on the integration from rectangular buckets, and the only significant differences observed between the authentic and non-original samples involved diethyl phthalate, galaxolide, benzyl salicylate, 2-ethylhexyl *trans*-4-methoxycinnamate and α-hexylcinnamaldehyde. The results for one selected fragrance were presented in Figure 5 and Figure 6 (for complete results see Supporting Information). The relative integration values from buckets showed significant differences in the means of the three group of samples. In the case of the counterfeit sample, and to a lesser extent inspired 2, a slight differences among points was observed. It was probably caused by the more complex processing of spectra, which contained large signals from solvents that may have led to signal distortion with low concentration in the aromatic region. As shown in Figure 6, diethyl phthalate, galaxolide, benzyl salicylate (and also isopropyl myristate in the case of *Light blue*) seemed to be the clearest indicators for discriminating authentic, inspired and counterfeit samples. Additionally, in the case of galaxolide, 2-ethylhexyl *trans*-4-methoxycinnamate and isopropyl myristate, the differentiation between authentic and inspired samples was not possible. The mean integration value for 2-ethylhexyl *trans*-4-methoxycinnamate allowed the authentic sample to be discriminated from the counterfeit, while in the case of the inspired samples, the differences were not statistically significant. On the basis of the α-hexylcinnamaldehyde, the differentiation between the authentic and counterfeit, as well as between the counterfeit and inspired 2 and 3, proved possible.

The higher level of 2-ethylhexyl *trans*-4-methoxycinnamate was observed in the case of authentic *Rush, Euphoria* and *Si* samples, while a lower content was observed for inspired and counterfeit samples. Moreover, the inspired sample of *Good girl* fragrance was characterized by a higher level of 2-ethylhexyl *trans*-4-methoxycinnamate in comparison to the authentic and counterfeit samples. The counterfeit samples of *J’adore, Euphoria, Rush* and *Si* were characterized by a higher level of benzyl salicylate. However, only in the case of *Rush* fragrances was it able to differentiate not only authentic and inspired samples from counterfeit but also authentic from inspired. The counterfeit and inspired samples of *Euphoria* showed higher integrations from diethyl phthalate, while in the case of *Si, Rush* and *J’adore* its higher content was only observed in counterfeit samples. Moreover, the counterfeit samples of *Euphoria, J’adore, Rush*, and *Good girl* demonstrated a higher level of hexyl cinnamal. On the other hand, a high concentration of galaxolide was observed in counterfeit samples of *Good girl, Euphoria,* and *J’adore,* while its content in authentic and inspired samples was at a comparable level (except for the inspired 5 sample of *J’adore*). These results are presented in the Supporting Information.

The most common methods used in the perfume laboratory, such as GC or LC chromatography, have the disadvantages of being time consuming, destructive, and not environmentally sound because of their use of solvents [26]. On the other hand, NMR spectroscopy is non-destructive, requires a small amount of solvent and is an excellent tool for the precise structural characterization of a pure compound, but for mixtures, overlapping signals appear to be the main issue. This methodology not only minimizes analysis time and cost but also provides adequate identification of fragrance components, such as non-volatile ingredients with low thermostability, that cannot be identified by GC–MS. Our results indicated that combination of ^1^H NMR spectroscopy with principal component analysis offers the possibility for the simultaneous identification of counterfeit samples without the need for signal identification. Therefore, the attractiveness of our method is high and might find further applications because the composition of the analysed mixture is not required. However, the information on the composition of a given product is available in the raw data and can be accessed if needed. Moreover, this methodology can be used in laboratories equipped with spectrometers with proton frequencies between 400 and 700 MHz.

## 3. Materials and Methods

### 3.1. Samples Preparation

In the present work, the eight authentic samples of perfumes (Dolce & Gabbana *Light Blue* (*n* = 2), G. Armani *Si*, C. Herrera *Good girl*, Gucci *Rush*, Dior *J’adore* (*n* = 2), and C. Klein *Euphoria*), six counterfeits and 14 inspired perfumes were analyzed (Table 1). The counterfeit and inspired samples were purchased over the Internet. Inspired samples were purchased from Yodeyma Paris, FM World, Refan and Eyfel. All samples for NMR measurements were prepared in the same way. A volume of 50 μL of analysed perfumery product was dissolved in 600 μL of deuterated chloroform (CDCl_3_ 99.8% D) with 0.03% (*v*/*v*) tetramethylsilane (TMS) as an internal standard. Five repetitions of a ^1^H NMR experiment were performed for each NMR tube.

### 3.2. NMR Measurements

All ^1^H NMR spectra were recorded using a Bruker Avance II Plus 16.4 T spectrometer (Bruker BioSpin, Rheinstetten, Germany) operating at the ^1^H frequency of 700.08 MHz The instrument was equipped with a 5 mm broadband BBI probe. ^1^H NMR spectra were acquired with a calibrated 90° pulse for 32 scans collecting 64 K data points over a spectral width of 14,097 Hz (zg, Bruker, Germany). The repetition time of 8.27 s, including a relaxation delay of 6 s, was calculated as 7 T_1_ of the longest relaxation time to ensure complete magnetization recovery. An exponential line broadening of 0.05 Hz was applied to the raw data prior to Fourier transformation. All samples were run at 300 K. The spectra were calibrated at 0 ppm from the TMS peak, which was used as a chemical shift standard. All spectral regions were individually corrected using a fifth-order baseline function.

### 3.3. Statistical Analysis

Statistical analysis was performed using AMIX 3.9.14 (Bruker, Rheinstetten, Germany) and OriginPro 2020 9.7.0.188 (OriginLab Northampton, MA, USA). The variation of the data was explored by principal component analysis (PCA), which was used for unsupervised pattern recognition, allowing the observation of trends and similarities between samples. The spectral region from 0 to 8.5 ppm of ^1^H NMR spectra was chosen as the input data for PCA analysis. Prior to chemometric analysis the NMR spectra were manually phased, the baseline was corrected and each spectrum calibrated to the TMS signal at 0 ppm. AMIX software was used to segment the NMR spectra into buckets. The width of the buckets was user-defined and equal to 0.05 ppm for ^1^H NMR data. The spectra were normalized to the total sum of the integrals of all the buckets. A one-way ANOVA was performed using the OriginPro 2020 to determine significant differences in compound levels. For an easier chemometric comparison, rectangular bucket integrals were used as input for one-way ANOVA analysis. The Tukey test was performed to reveal pair-wise differences between means (*p* < 0.05).

## 4. Conclusions

The first approach to a rapid and reliable discrimination of perfumes based on proton nuclear magnetic resonance spectroscopy was demonstrated. This method is characterized by a very fast and simple sample preparation that allows the discrimination of authentic, counterfeit and inspired perfumes on the basis of the synergic combination of ^1^H NMR spectroscopy and chemometric techniques. Our research showed that ^1^H NMR fingerprints may serve as an alternative method to identify counterfeit products. However, the number of samples was a limitation of the study, but in our opinion it indicated an overall trend in the discrimination of perfume. Furthermore, the small differences observed for two authentic *J’adore* samples suggest that the comparison of authentic samples from different batches is necessary. We believe that the developed method possesses great potential, and further studies on the application of NMR spectroscopy in such studies are ongoing in our laboratory.

## Figures and Tables

**Table 1:** A list of the considered perfumes with their denotations.

	*Light Blue*	*J’adore*	*Rush*	*Euphoria*	*Good Girl*	*Si*
Authentic	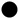	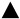	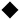	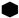	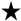	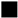
Inspired 1	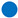	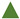	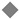	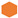	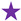	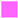
Inspired 2	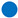	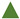	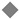			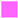
Inspired 3	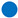	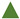				
Inspired 4		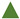				
Inspired 5		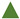				
Counterfeit	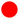	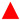	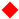	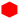	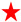	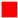

## Data Availability

The data presented in this study are available in the article and in the Supplementary Material.

## Supplementary Materials

The following are available online. Figure S1: The comparison of the means integration values of ^1^H NMR signals for authentic, inspired and counterfeited *Light Blue* samples obtained by ANOVA. (Tukey test, *p* < 0.05). Figure S2: The comparison of the means integration values of ^1^H NMR signals for authentic, inspired and counterfeited *Good girl* samples obtained by ANOVA. (Tukey test, *p* < 0.05). Figure S3: The comparison of the means integration values of ^1^H NMR signals for authentic, inspired and counterfeited *Good girl* samples obtained by ANOVA. (Tukey test, *p* < 0.05). Figure S4: The comparison of the means integration values of ^1^H NMR signals for authentic, inspired and counterfeited *Si* samples obtained by ANOVA. (Tukey test, *p* < 0.05). Figure S5: The comparison of the means integration values of ^1^H NMR signals for authentic, inspired and counterfeited *Si* samples obtained by ANOVA. (Tukey test, *p* < 0.05). Figure S6: The comparison of the means integration values of ^1^H NMR signals for authentic, inspired and counterfeited *Rush* samples obtained by ANOVA. (Tukey test, *p* < 0.05). Figure S7: The comparison of the means integration values of ^1^H NMR signals for authentic, inspired and counterfeited *Rush* samples obtained by ANOVA. (Tukey test, *p* < 0.05). Figure S8: The comparison of the means integration values of ^1^H NMR signals for authentic, inspired and counterfeited *J’adore* samples obtained by ANOVA. (Tukey test, *p* < 0.05). Figure S9: The comparison of the means integration values of ^1^H NMR signals for authentic, inspired and counterfeited *J’adore* samples obtained by ANOVA. (Tukey test, *p* < 0.05). Figure S10: The comparison of the means integration values of ^1^H NMR signals for authentic, inspired and counterfeited *Euphoria* samples obtained by ANOVA. (Tukey test, *p* < 0.05). Figure S11: The comparison of the means integration values of ^1^H NMR signals for authentic, inspired and counterfeited *Euphoria* samples obtained by ANOVA. (Tukey test, *p* < 0.05). Figure S12: ^1^H NMR spectra of authentic, inspired and counterfeited samples of *Light blue* in spectral region from 0 to 11 ppm. Figure S13: Expanded region of ^1^H NMR spectrum of inspired *Light blue* sample in the region of chemical shift from 3.85 ppm and to 5.15 ppm. Figure S14: ^1^H NMR spectra of authentic, inspired and counterfeited samples of *Good girl* in spectral region from 0 to 11 ppm. Figure S15: ^1^H NMR spectra of authentic, inspired and counterfeited samples of *Si* in spectral region from 0 to 11 ppm. Figure S16: ^1^H NMR spectra of authentic, inspired and counterfeited samples of *Rush* in spectral region from 0 to 11 ppm. Figure S17: ^1^H NMR spectra of authentic, inspired and counterfeited samples of *J’adore* in spectral region from 0 to 11 ppm. Figure S18: ^1^H NMR spectra of authentic, inspired and counterfeited samples of *Euphoria* in spectral region from 0 to 11 ppm. Figure S19: PCA loading plot generated from the ^1^H NMR spectra from the authentic perfume and counterfeited and inspired samples for the range of chemical shifts from 0 to 10 ppm. Figure S20: The comparison of the buckets from NMR spectra for two authentic samples of *Light blue*. Figure S21: The comparison of the buckets from NMR spectra for two authentic samples of *J’adore*. Table S1: The main compounds assigned in ^1^H NMR spectra of perfume samples, their diagnostic ^1^H signals, chemical shifts δ_H_ and multiplicities.

## Abbreviations

GC–MS	gas chromatography coupled with mass spectrometry
EOS	electronic olfactory system
DOSY	diffusion ordered spectroscopy
PCA	principal component analysis
TMS	tetramethylsilane

## References

[1] Dronova O., Smagorinskiy B.P., Yastrebov V., Kravets A.G. (2019). Counteraction to e-commerce crimes committed with the use of online stores. Big Data-Driven World: Legislation Issues and Control. Technologies.

[2] Cano M., Borrego V., Roales J., Idigoras J., Lopes-Costa T., Mendoza P., Pedrosa J.M. (2011). Rapid discrimination and counterfeit detection of perfumes by an electronic olfactory system. Sens. Actuators B Chem..

[3] Abedi G., Talebpour Z., Jamechenarboo F. (2018). The survey of analytical methods for sample preparation and analysis of fragnances in cosmetics and personal care products. Trends Anal. Chem..

[4] Heydorn S., Menné T., Johansen J.D. (2003). Fragrance allergy and hand eczema—A review. Contact Derm..

[5] Steinemann A. (2016). Fragranced consumer products: Exposures and effects from emissions. Air Qual. Atmos. Health.

[6] Basketter D.A., Lemoine S., McFadden J.P. (2015). Skin sensitisation to fragrance ingredients: Is there a role for household cleaning/maintenance products?. Eur. J. Dermatol..

[7] Fortineau A.D. (2004). Chemistry Perfumes Your Daily Life. J. Chem. Educ..

[8] Deska M., Girek T., Herman B. (2016). Środki konserwujące w preparatach i kosmetycznych i bezpieczeństwo ich stosowania. Prace Nauk. Akad. Im. Jana Długosza W Częstochowie Tech. Inform. Inżynieria Bezpieczeństwa.

[9] Rudzki E., Parapura K., Czubalska M. (2002). Alergia na perfumy. Alerg. Astma Immunol..

[10] van Asten A. (2002). The importance of GC and GC–MS in perfume analysis. Trends Analyt. Chem..

[11] Debonneville C., Chaintreau A. (2004). Quantitation of suspected allergens in fragrances. Part II: Evaluation of comprehensive gas chromatography conventional mass spectrometry. J. Chromatogr. A.

[12] Casabianca H., Graff J.B., Jame P., Perrucchietti C., Chastrette M. (1995). Application of hyphenated techniques to the Cromatographic authentication of flavors in food-products and perfumes. J. High. Resolut. Chromatogr..

[13] Cabaleiro N., de la Calle I., Bendicho C., Lavilla I. (2012). Fast screening of terpenes in fragrance-free cosmetics by fluorescence quenching on a fluoresceine bovine serum albumin probe confined in a drop. Anal. Chim. Acta.

[14] Hoffman R.E., Arzuan H., Pemberton C., Aserin A., Garti N. (2008). High-resolution NMR “chromatography” using a liquids spectrometer. J. Magn. Reson..

[15] Pemberton C., Hoffman R.E., Aserin A., Garti N. (2011). NMR chromatography usingmicroemulsion systems. Langmuir.

[16] Talzi V.P. (2006). A ^13^C and ^1^H NMR analysis of perfumes. Russ. J. Appl. Chem..

[17] Haddad R., Catharino R.R., Marques L.A., Eberlin M.N. (2008). Perfume fingerprinting by easy ambient sonic-spray ionization mass spectrometry: Nearly instantaneous typification and counterfeit detection. Rapid Commun. Mass Spectrom..

[18] Chingin K., Gamez G., Chen H., Zhu L., Zenobi R. (2008). Rapid classification of perfumes by extractive electrospray ionization mass spectrometry (EESI-MS). Rapid Commun. Mass Spectrom..

[19] Wilson A.D., Baietto M. (2009). Applications and advances in electronic-nose technologies. Sensors.

[20] Poprawski J., Boilot P., Tetelin F. (2006). Counterfeiting and quantification using an electronic nose in the perfumed cleaner industry. Sens. Actuators B Chem..

[21] Gherghel S., Morgan R.M., Blackman C.S., Karu K., Parkin I.P. (2016). Analysis of transferred fragrance and its forensic implications. Sci. Justice.

[22] Gherghel S., Morgan R.M., Arrebola-Liébanas J., Romero-González R., Blackman C.S., Garrido-Frenich A., Parkin I.P. (2018). Development of a HS-SPME/GC-MS method for the analysis of volatile organic compounds from fabrics for forensic reconstruction applications. Forensic Sci. Int..

[23] Gherghel S., Morgan R.M., Arrebola-Liébanas J.F., Blackman C.S., Parkin I.P. (2019). Fragrance transfer between fabrics for forensic reconstruction applications. Sci. Justice.

[24] Godinho R.B., Santos M.C., Poppi R.J. (2016). Determination of fragrance content in perfume by Raman spectroscopy and multivariate calibration. Spectrochim. Acta A Mol. Biomol. Spectrosc..

[25] Borowiecka J., Wesołowski W. (2010). Analiza składników wyrobów perfumeryjnych z rodziny owocowej przeznaczonych dla kobiet techniką GC/MS. Bromat. Chem. Toksykol..

[26] Bombarda I., Dupuy N., Le Van Da J.P., Gaydou E.M. (2008). Comparative chemometric analyses of geographic origins and compositions of lavandin var. Grosso essential oils by mid infrared spectroscopy and gas chromatography. Anal. Chim. Acta.

