# Supplementary material for: The First Application of 1H NMR Spectroscopy for the Assessment of the Authenticity of Perfumes
Source: Molecules. 2021 May 22;26(11):3098. doi: 10.3390/molecules26113098 (PMC8196850; doi:10.3390/molecules26113098)
Supplement: Supplementary file 1 [file molecules-26-03098-s001.zip › molecules-1229885-SI.pdf]

Supporting information

The first application of ^{1}H NMR spectroscopy for the assessment of the authenticity of perfumes

Barbara Pacholczyk-Sienicka,[a*] Grzegorz Ciepielowski,[a] Łukasz Albrecht[a]

[a]*Institute of Organic Chemistry, Faculty of Chemistry, Lodz University of Technology, Zeromskiego 116, 90-924 Lodz, Poland*

Table of contents

1. Comparison of integration values of ^{1}H NMR signals 2

2. Representative ^{1}H NMR spectra of the authentic, inspired and counterfeited samples. 13

3. Table with main compounds assigned in ^{1}H NMR spectra of perfume samples 20

4. PCA analysis conducted for full spectrum bucket tables. 25

5. Comparison of the bucket tables from NMR spectra for two authentic samples. 26

1. Comparison of integration values of ^{1}H NMR signals

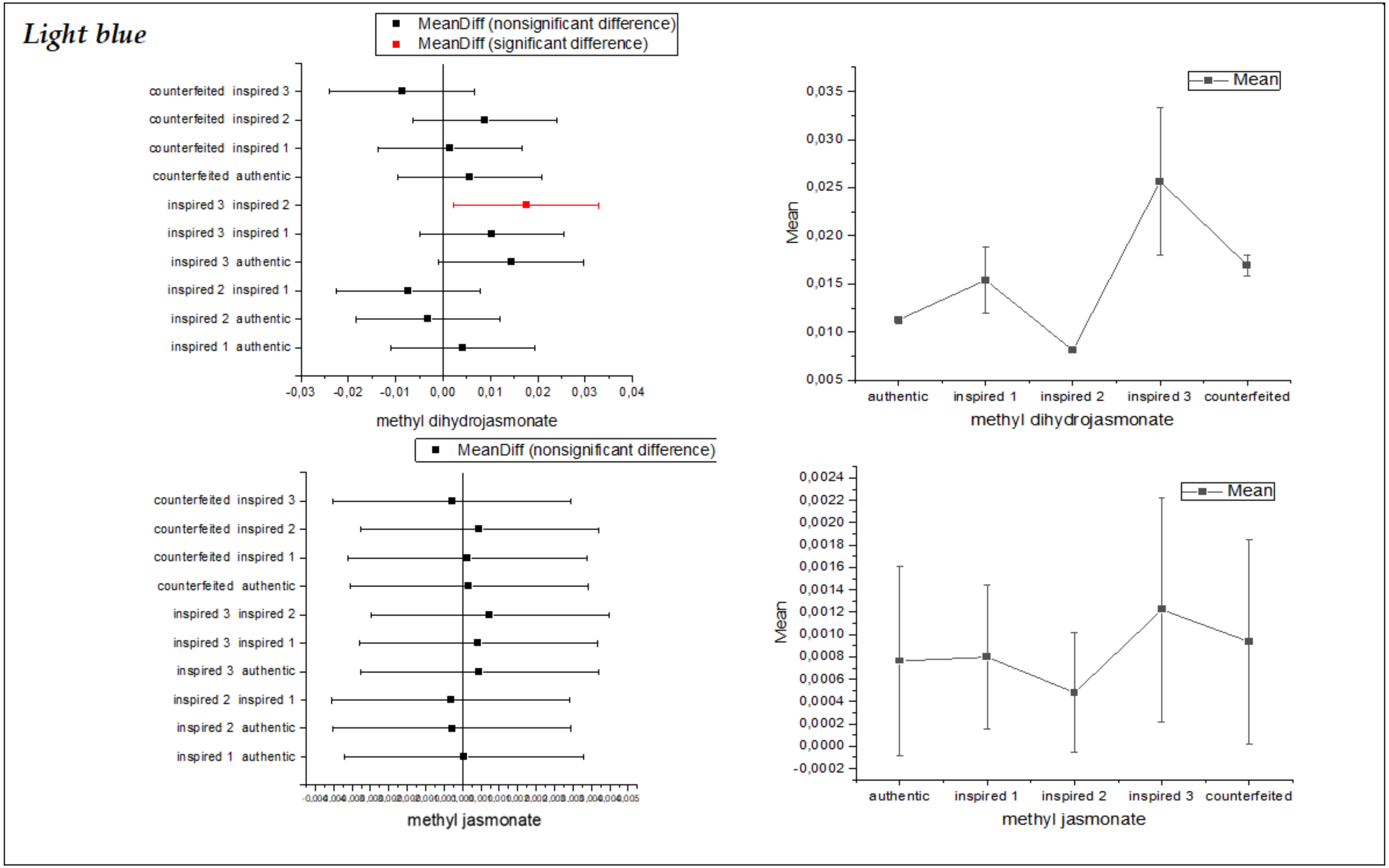

Figure S1 The comparison of the means integration values of ^{1}H NMR signals for authentic, inspired and counterfeited *Light Blue* samples obtained by ANOVA. (Tukey test, $p<0.05$).

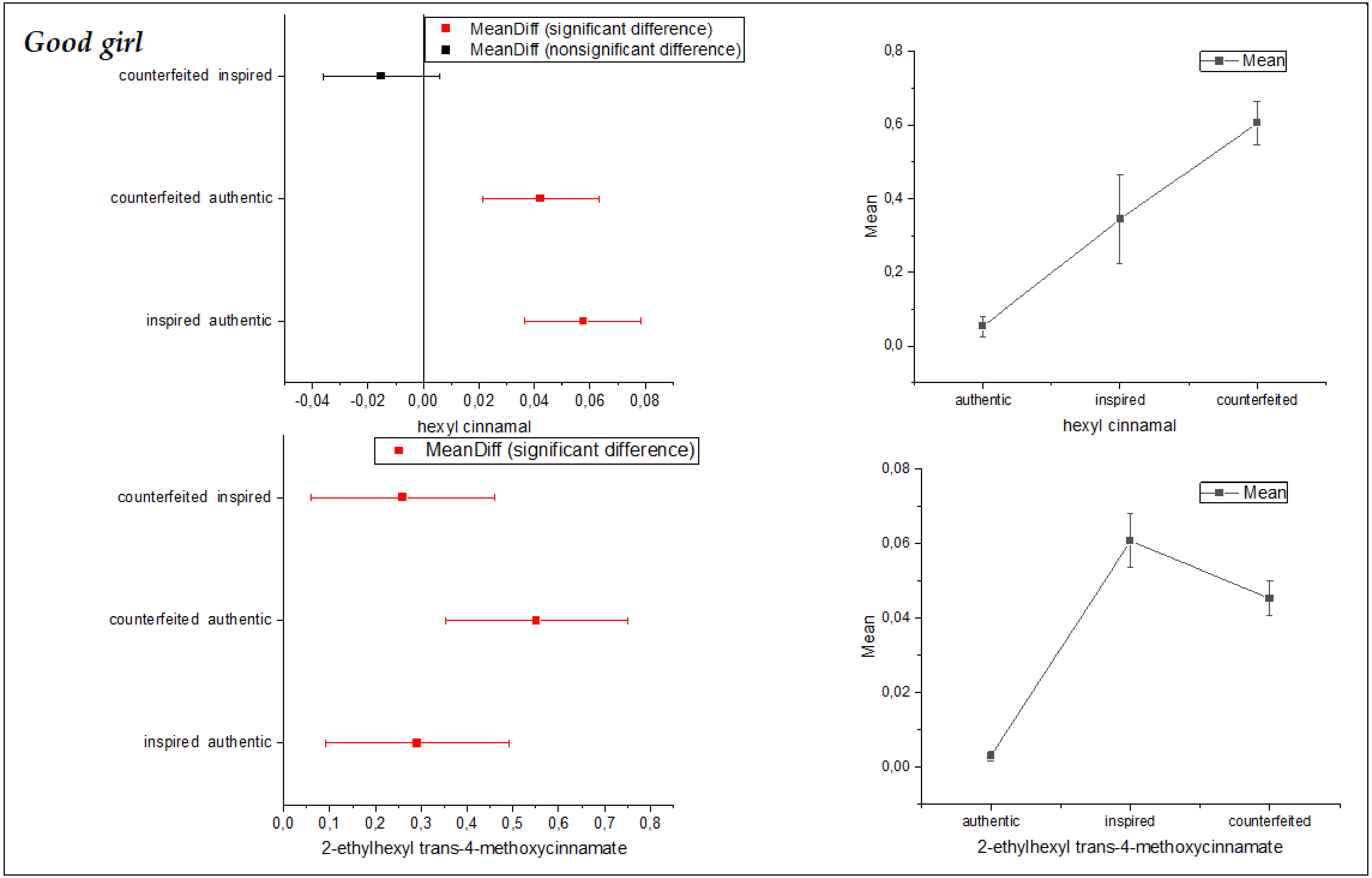

Figure S2 The comparison of the means integration values of ^{1}H NMR signals for authentic, inspired and counterfeited *Good girl* samples obtained by ANOVA. (Tukey test, $p<0.05$).

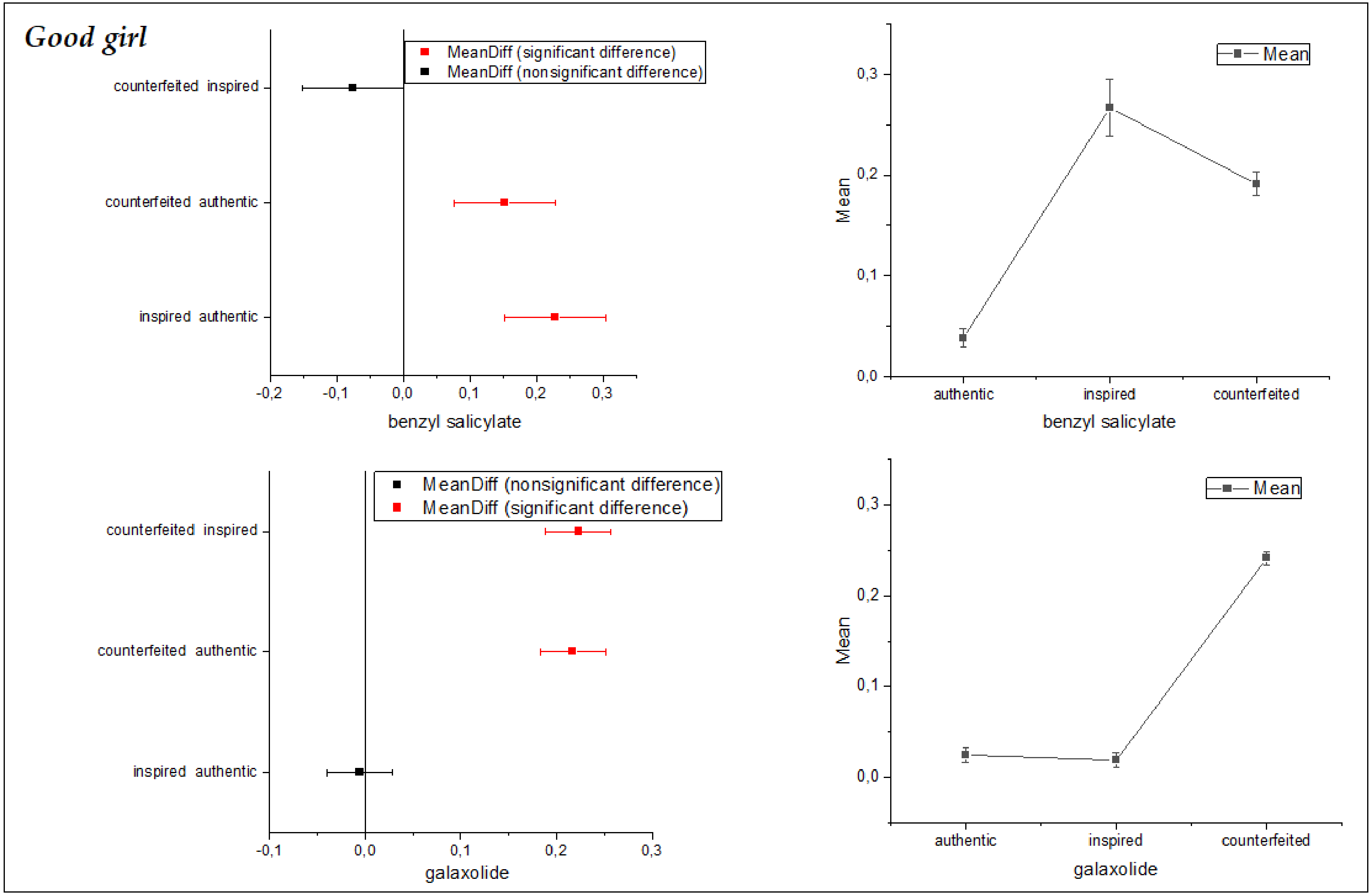

Figure S3 The comparison of the means integration values of ^{1}H NMR signals for authentic, inspired and counterfeited *Good girl* samples obtained by ANOVA. (Tukey test, $p<0.05$).

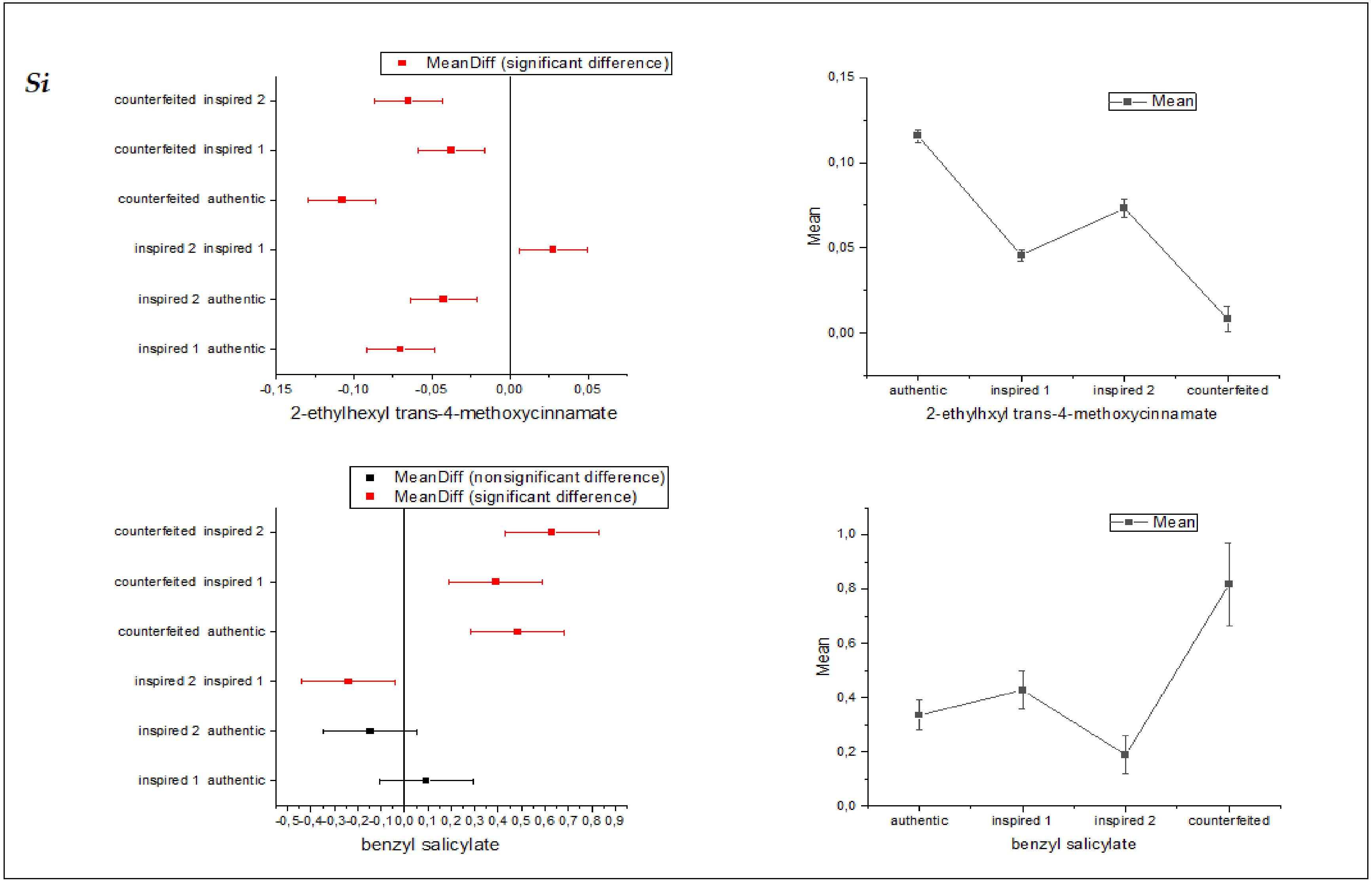

Figure S4 The comparison of the means integration values of ^{1}H NMR signals for authentic, inspired and counterfeited *Si* samples obtained by ANOVA. (Tukey test, $p<0.05$).

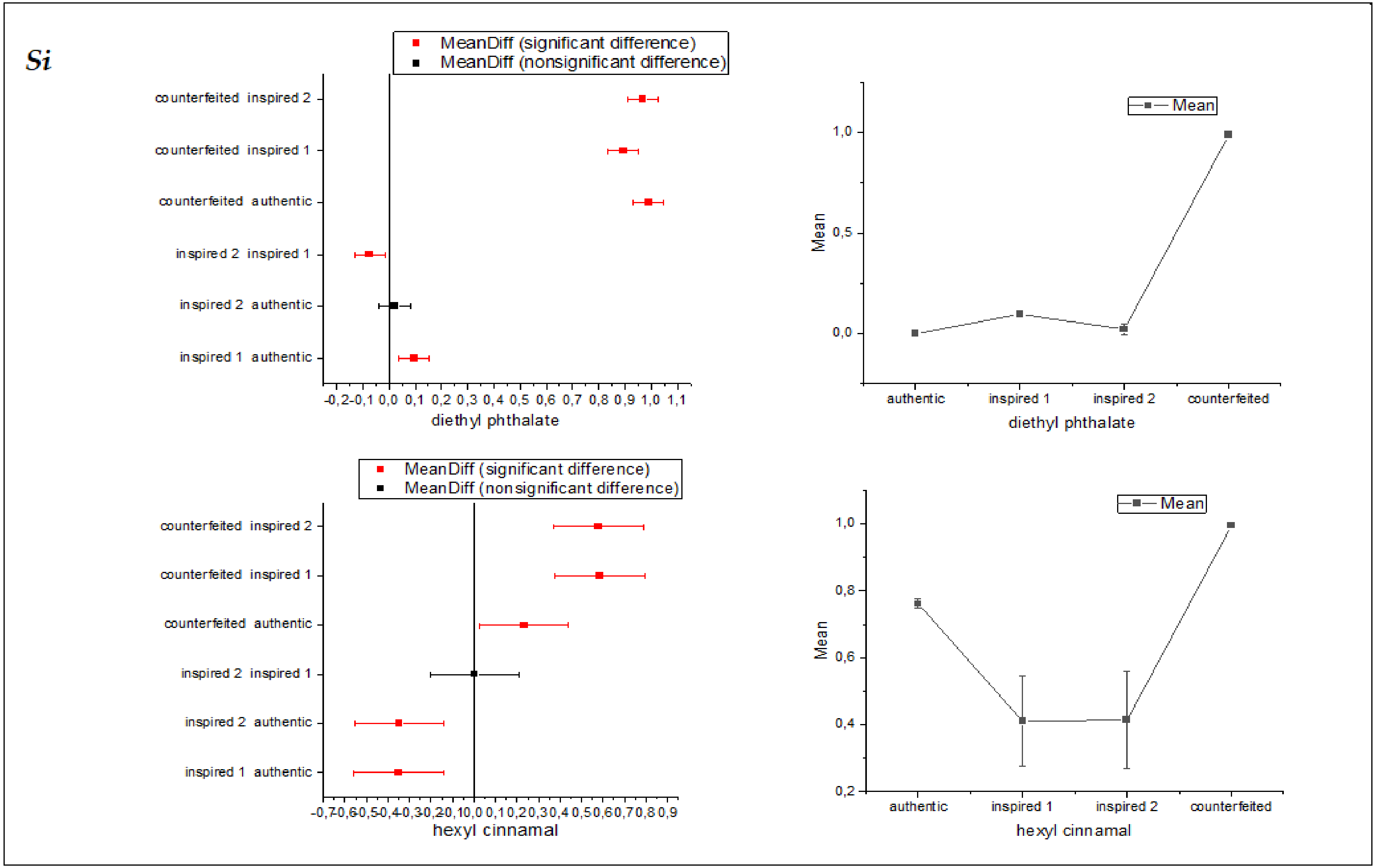

Figure S5 The comparison of the means integration values of ^{1}H NMR signals for authentic, inspired and counterfeited *Si* samples obtained by ANOVA. (Tukey test, $p<0.05$).

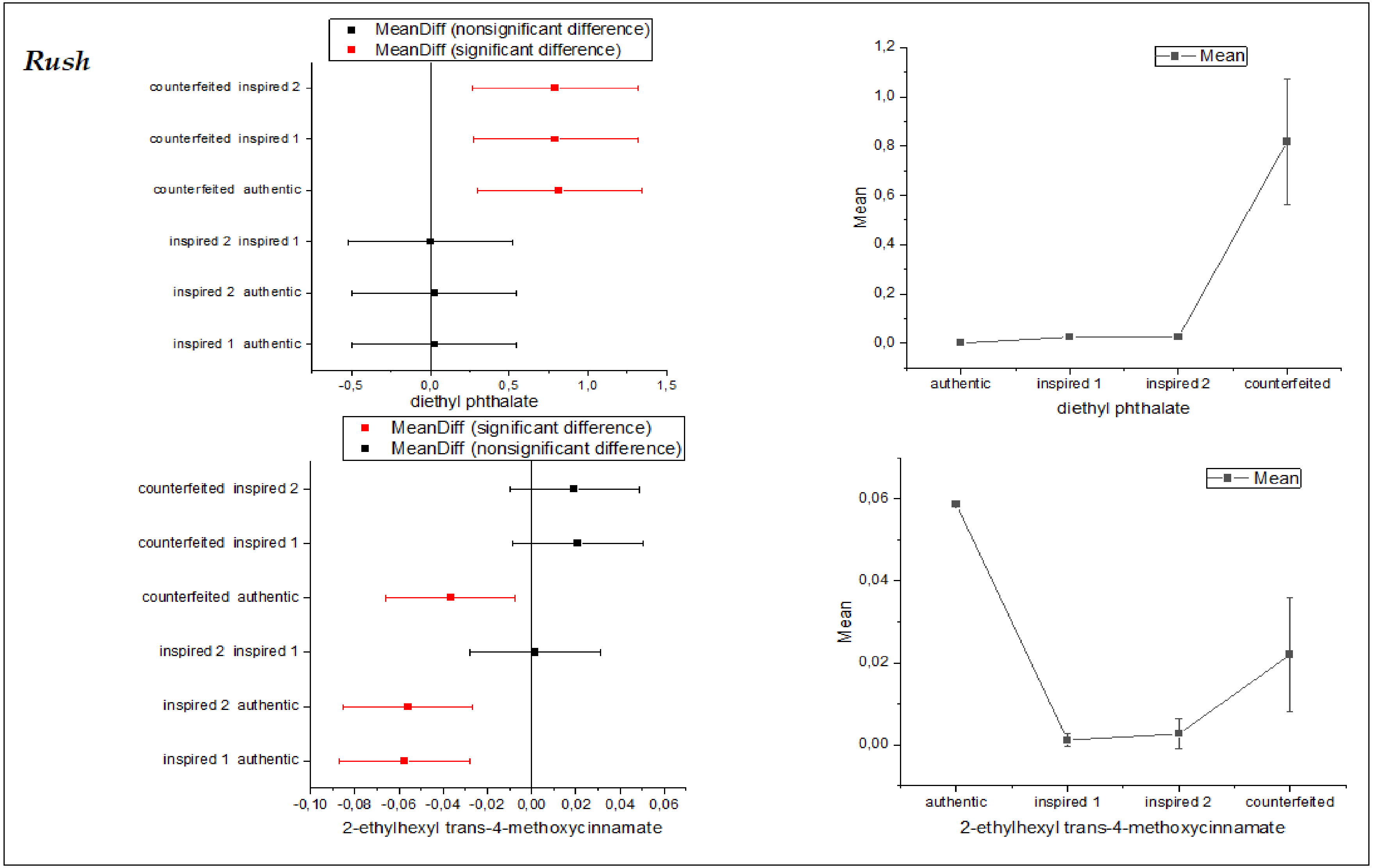

Figure S6 The comparison of the means integration values of ^{1}H NMR signals for authentic, inspired and counterfeited *Rush* samples obtained by ANOVA. (Tukey test, $p<0.05$).

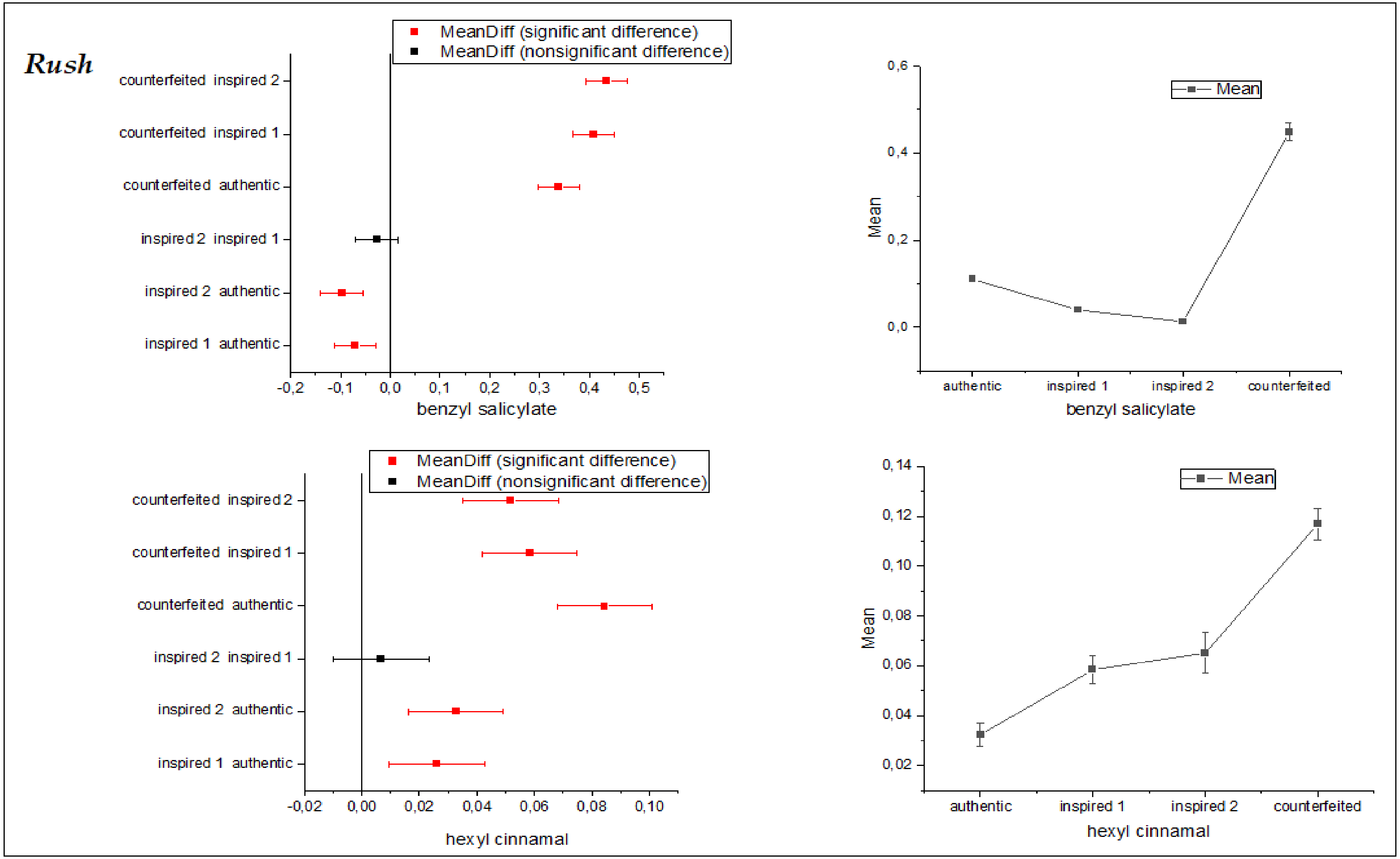

Figure S7 The comparison of the means integration values of ^{1}H NMR signals for authentic, inspired and counterfeited *Rush* samples obtained by ANOVA. (Tukey test, $p<0.05$).

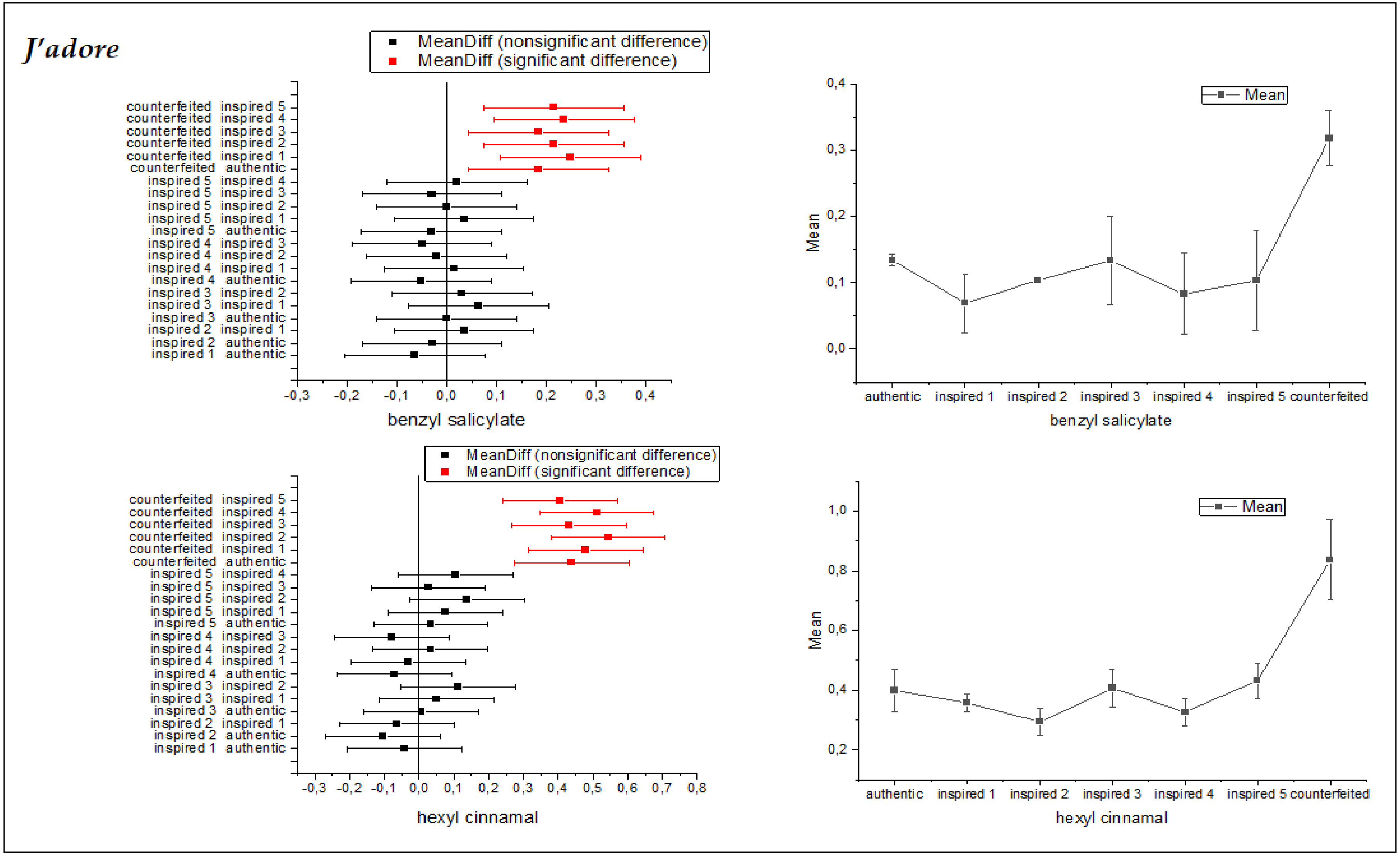

Figure S8 The comparison of the means integration values of ^{1}H NMR signals for authentic, inspired and counterfeited *J'adore* samples obtained by ANOVA. (Tukey test, $p<0.05$).

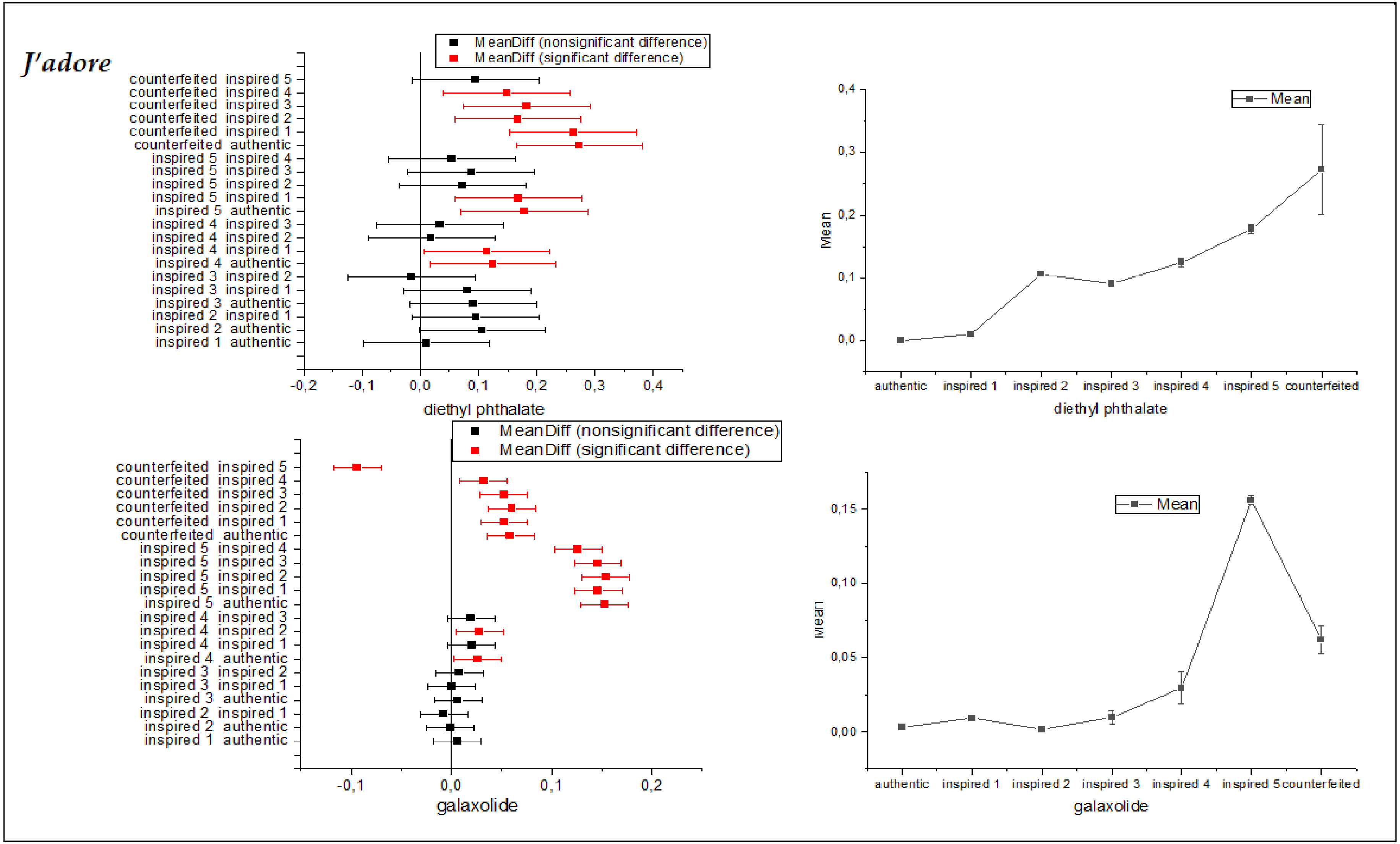

Figure S9 The comparison of the means integration values of ^{1}H NMR signals for authentic, inspired and counterfeited *J'adore* samples obtained by ANOVA. (Tukey test, $p<0.05$).

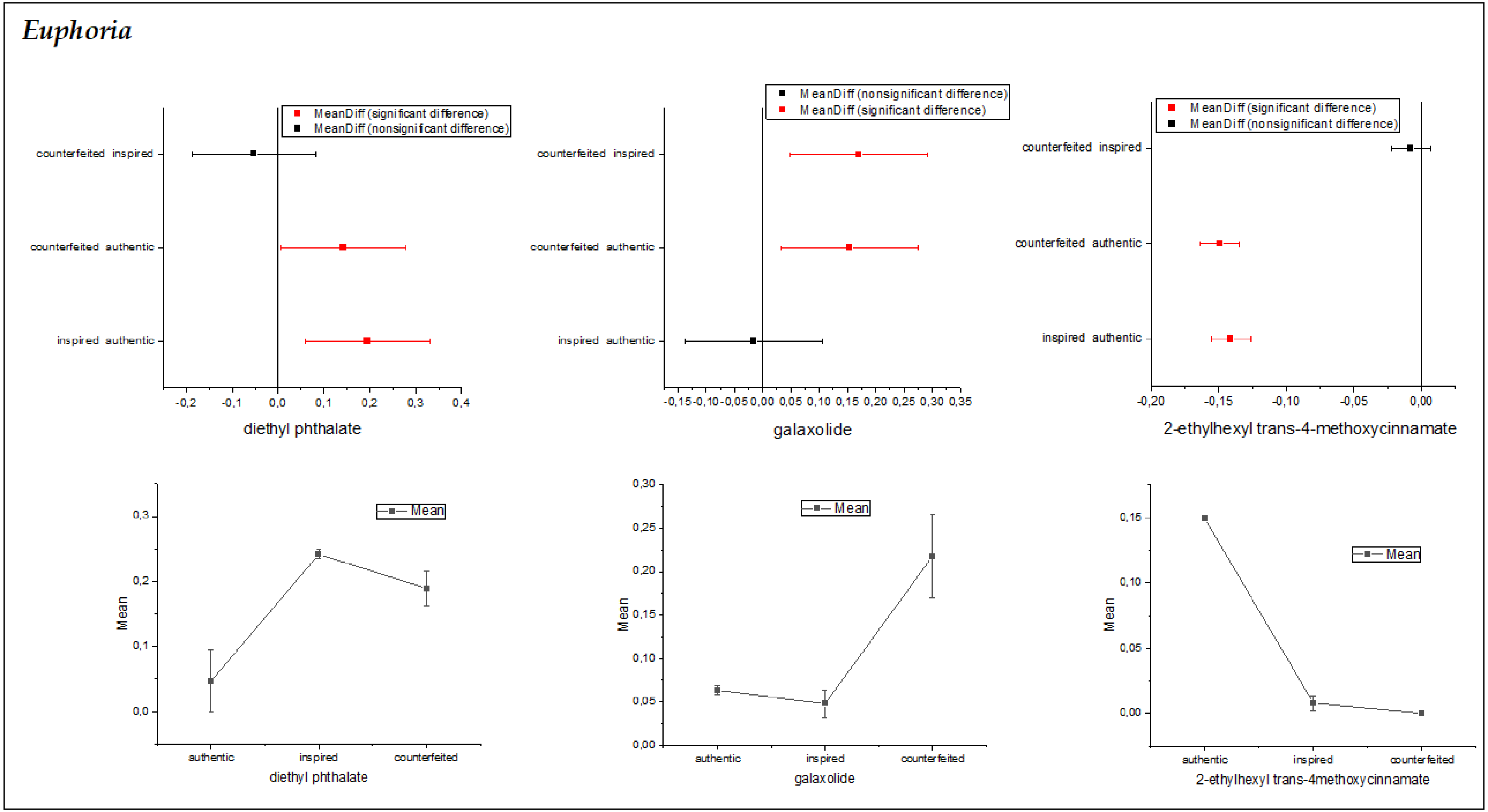

Figure S10 The comparison of the means integration values of ¹H NMR signals for authentic, inspired and counterfeited *Euphoria* samples obtained by ANOVA. (Tukey test, $p<0.05$).

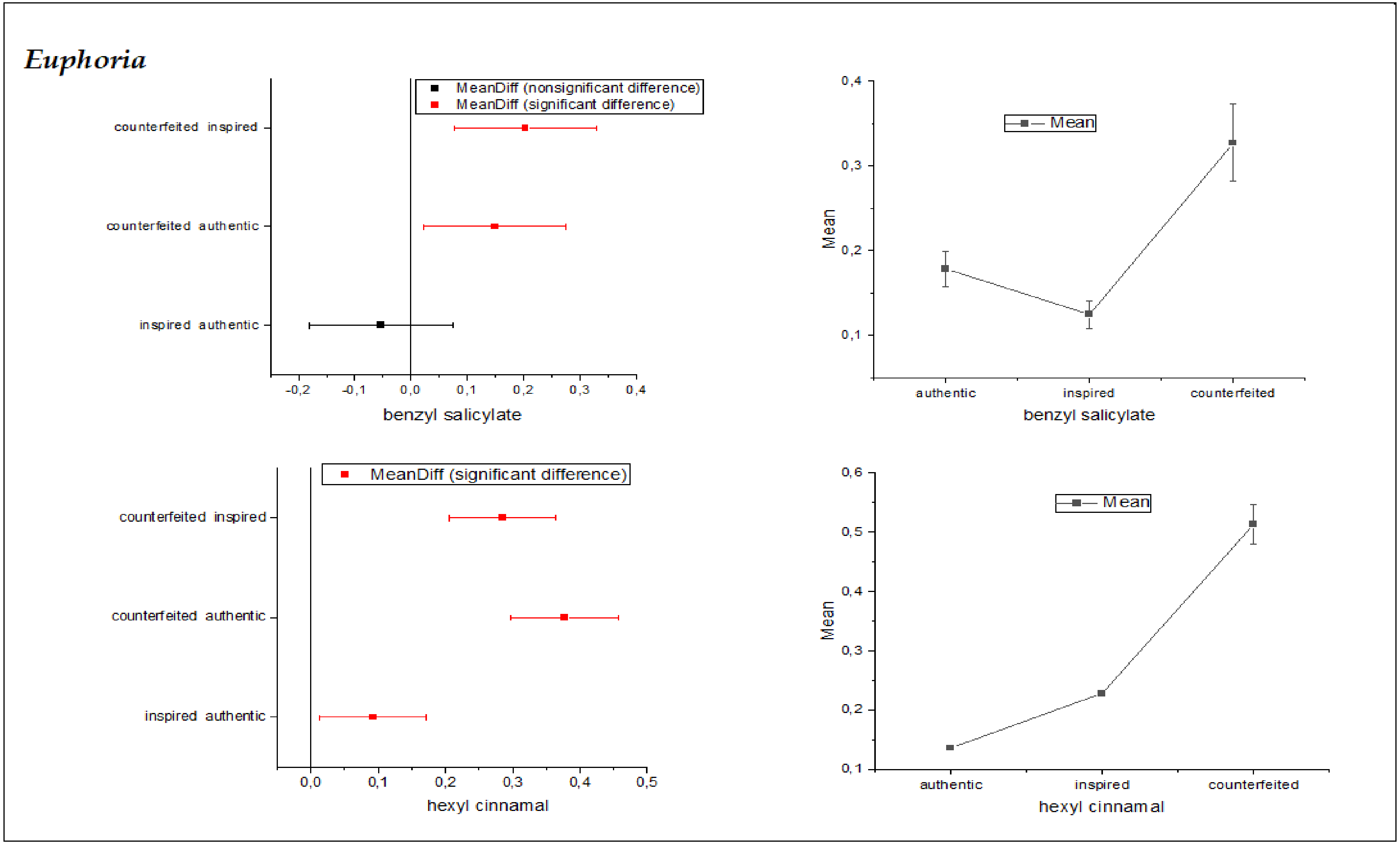

Figure S11 The comparison of the means integration values of ^{1}H NMR signals for authentic, inspired and counterfeited *Euphoria* samples obtained by ANOVA. (Tukey test, $p<0.05$).

2. Representative ^{1}H NMR spectra of the authentic, inspired and counterfeited samples.

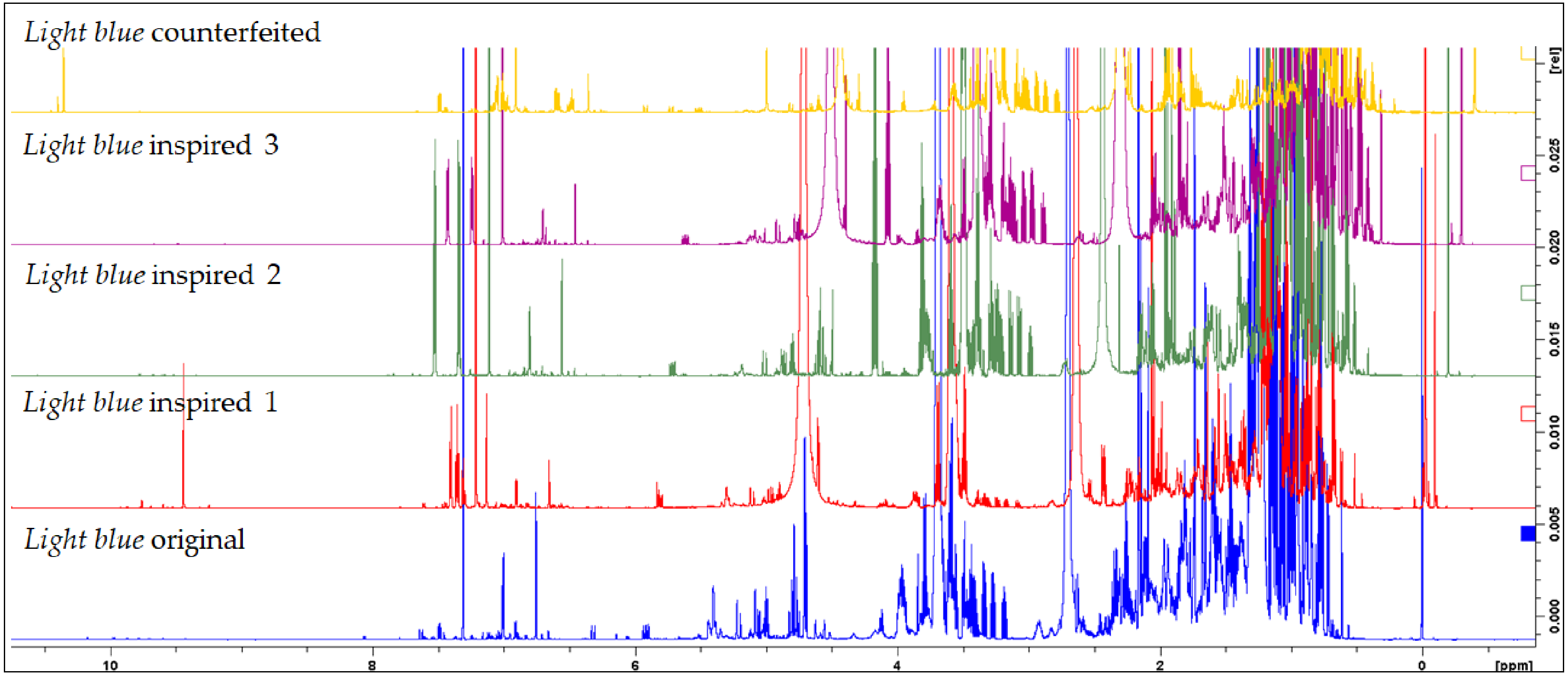

Figure S12 ^{1}H NMR spectra of authentic, inspired and counterfeited samples of *Light blue* in spectral region from 0 to 11 ppm.

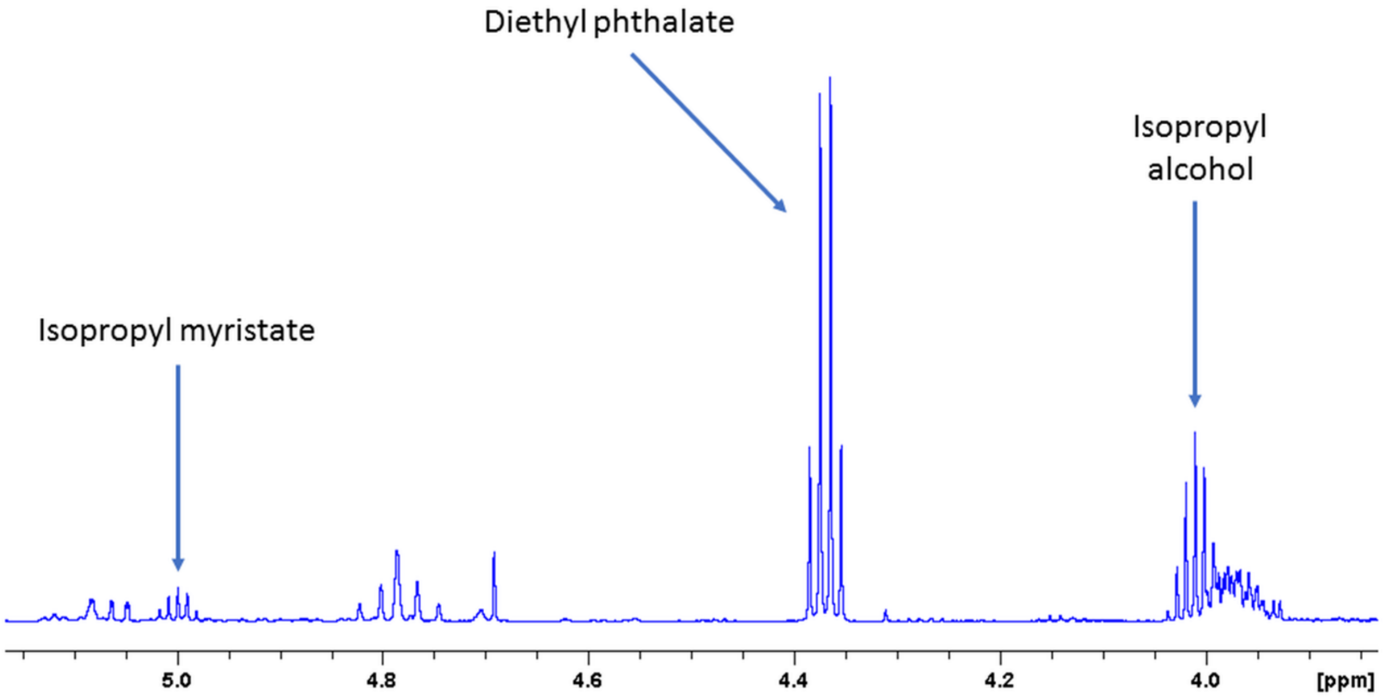

Figure S13. Expanded region of ^{1}H NMR spectrum of inspired *Light blue* sample in the region of chemical shift from 3.85 ppm and to 5.15 ppm.

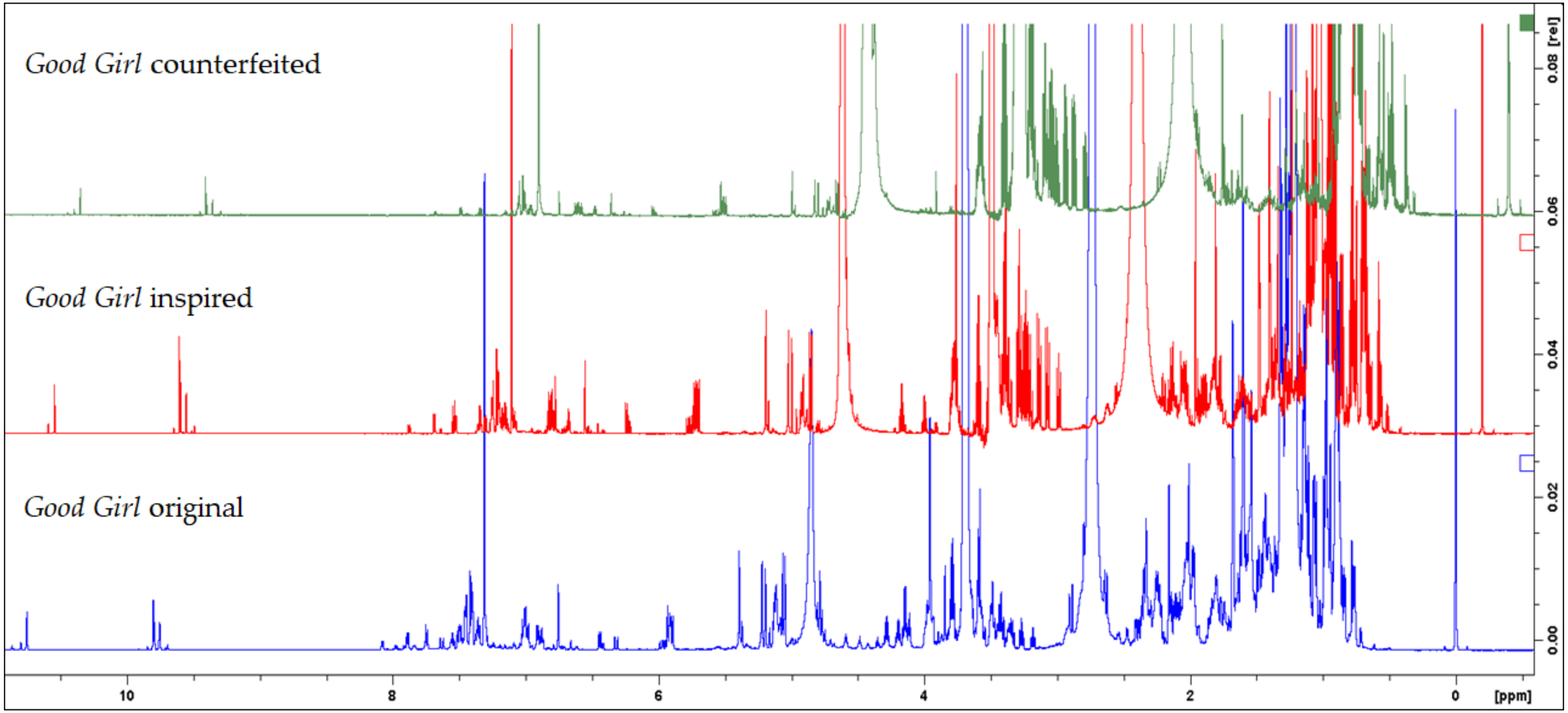

Figure S14 ^{1}H NMR spectra of authentic, inspired and counterfeited samples of *Good girl* in spectral region from 0 to 11 ppm.

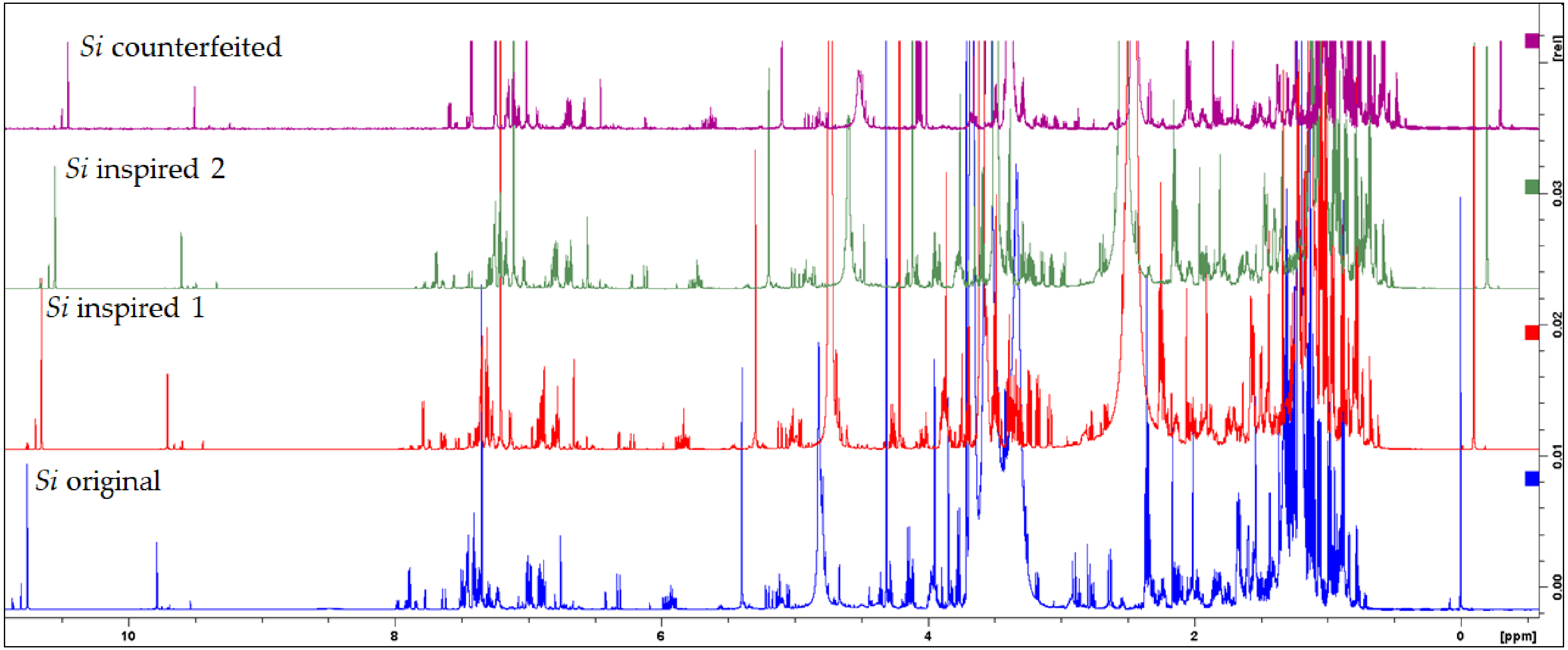

Figure S15 ^{1}H NMR spectra of authentic, inspired and counterfeited samples of *Si* in spectral region from 0 to 11 ppm.

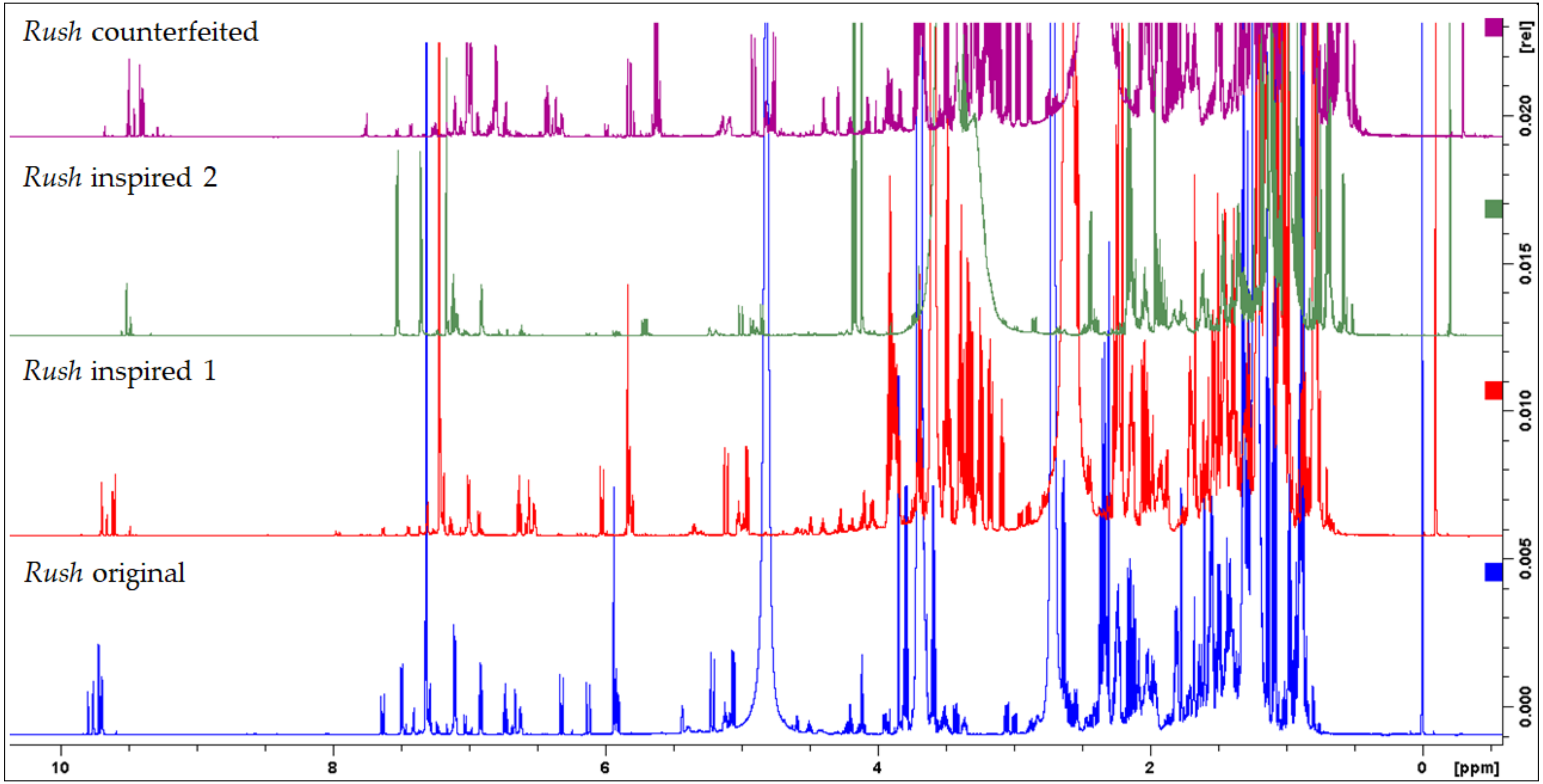

Figure S16 ¹H NMR spectra of authentic, inspired and counterfeited samples of *Rush* in spectral region from 0 to 11 ppm.

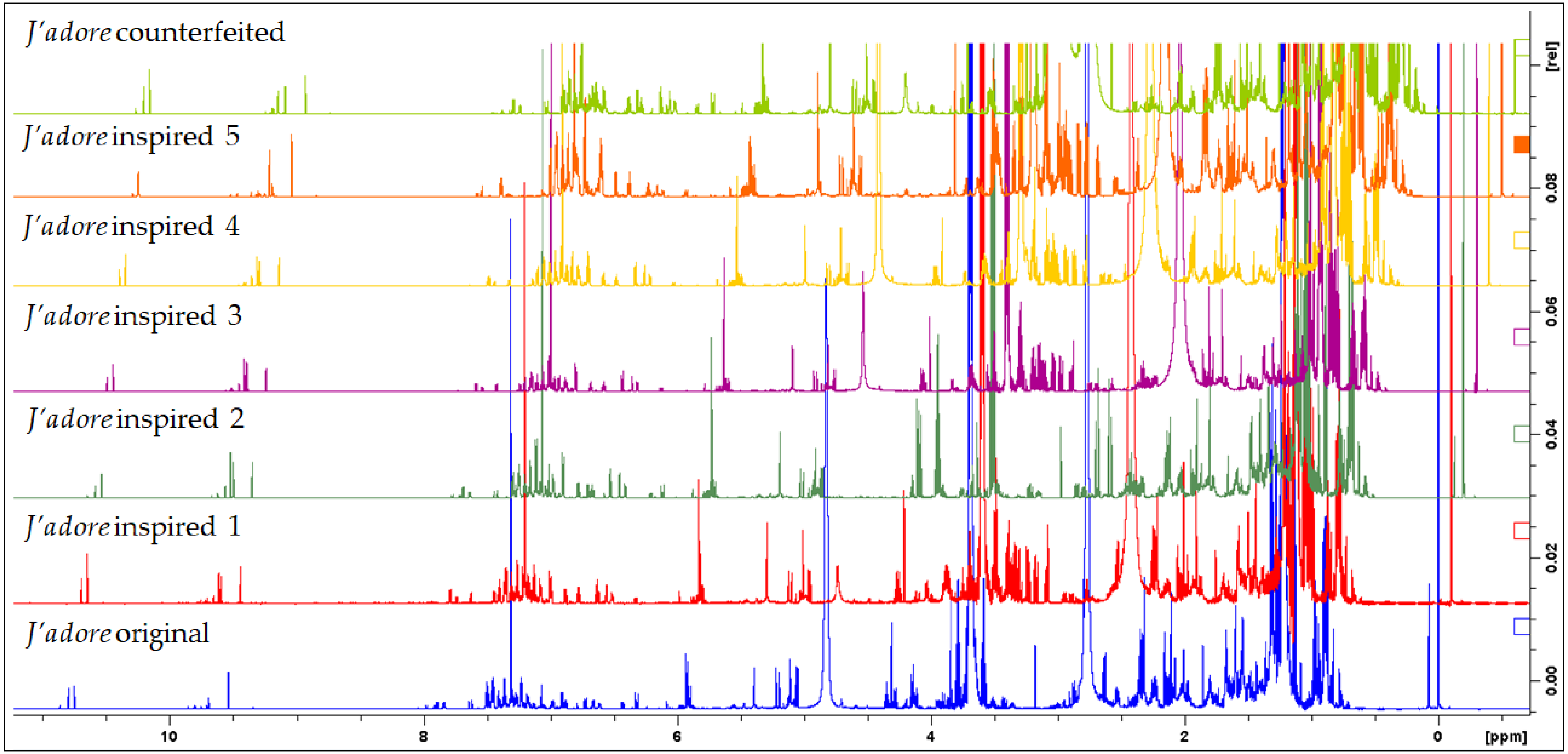

Figure S17 ^{1}H NMR spectra of authentic, inspired and counterfeited samples of *J'adore* in spectral region from 0 to 11 ppm.

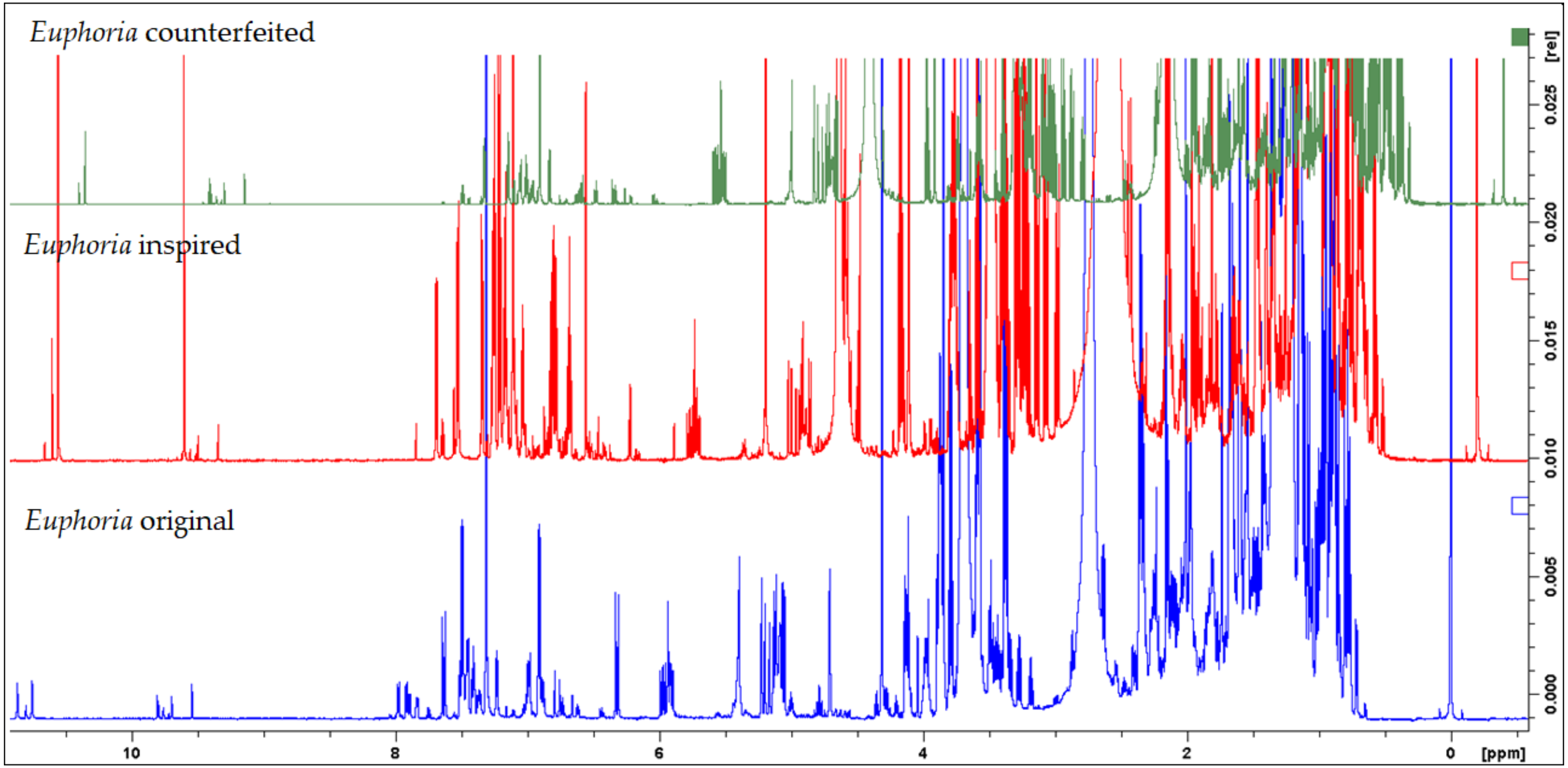

Figure S18 ^{1}H NMR spectra of authentic, inspired and counterfeited samples of *Euphoria* in spectral region from 0 to 11 ppm.

3. Table with main compounds assigned in ^{1}H NMR spectra of perfume samples

Table S1. The main compounds assigned in ^{1}H NMR spectra of perfume samples, their diagnostic ^{1}H signals, chemical shifts δ_H and multiplicities.

LP	Compound	Functional group	Chemical shift [ppm] (multiplet)
**1**	α-Hexyl cinnamaldehyde*	-C(O)H	9.53 (s)
		2xH_{Ar}	7.47 (d)
		2xH_{Ar}	7.42 (t)
		H_{Ar}	7.38 (t)
		CH=	7.17 (s)
**2**	Benzyl alcohol	5xH_{Ar}	7.20 -7.40(m)
		-CH_2-	4.54 (d)
**3**	Cinnamaldehyde	-C(O)H	9.69 (d)
		H_{Ar}	6.72 (dd)
		H_{Ar}	7.57 (m)
		H_{Ar}	7.44 (m)
**4**	β-pinene*	=CH_2	4.55 (m) and 4.62 (m)
**5**	Benzaldehyde	-C(O)H	10.02 (s)
		2xH_{Ar}	7.86 (d)
		H_{Ar}	7.60 (t)
		2xH_{Ar}	7.51 (t)
**6**	Benzyl benzoate	-CH_2-	5.35 (s)
		2xH_{Ar}	8.07 (d)
		H_{Ar}	7.55 (t)
		2xH_{Ar}	7.41 (t)
		2xH_{Ar}	7.3-7.5 (m)

LP	Compound	Functional group	Chemical shift [ppm] (multiplet)
**7**	Ethylene brasylate*	$-CH_2-$	4.31 (s)
**8**	Citral	-C(O)<u>H</u>	9.87 (d)
		=CH	5.81 (d)
**9**	Citronellal	-C(O)<u>H</u>	9.75 (t)
		-CH=	5.11 (m)
**10**	Methyl dihydrojasmonate*	$-CH_3$	3.63 (s)
**11**	Ethylvanillin	-C(O)<u>H</u>	9.85 (s)
		$-OCH_2-$	4.15 (q)
**12**	Vanillin	-C(O)<u>H</u>	9.83 (s)
**13**	Eugenol	$-OCH_3$	3.84 (s)
**14**	Diethyl phthalate*	$-CH_3$	1.37 (t)
		$-CH_2-$	4.37 (q)
		H_{Ar}	7.72 (dd)
		H_{Ar}	7.53 (dd)
**15**	Galaxolide*	H_{Ar}	6.76 (s)
		H_{Ar}	7.00 (s)
**16**	γ-Terpinene*	=CH-	5.43 (br.s)
		$-CH_2-$	2.60 (br.s)
**17**	Geraniol	$-CH_2-$	5.20 (dt)
**18**	Coumarin	=CH-	6.42 (d)
		C(O)O-	
**19**	Lilial	-C(O)H	9.67 (d)
		$-(CH_3)_3$	1.39 (s)
**20**	Linalool*	$H_2C=C\underline{H}-$	5.92 (m)

LP	Compound	Functional group	Chemical shift [ppm] (multiplet)
		$H_2C=CH-$	5.04 (dd) and 5.21 (dd)
**21**	Limonene*	$=CH_2$	4.71 (s)
		=CH-	5.40 (br.s)
**22**	Isopropyl myristate*	-CH-	5.00 (sp)
		$-(CH_3)_2$	1.23 (d)
**23**	Benzyl salicylate	$-CH_2-$	5.39 (s)
		H_{Ar}	6.88 (t)
		H_{Ar}	6.97 (d)
		H_{Ar}	7.32-7.5 (m)
		H_{Ar}	7.85 (d)
		-OH	10.75 (s)
**24**	Cyclohexene*	$-CH_2-$	5.66 (br.s)
**25**	Isopropyl alcohol	-CH-	4.07 (sp)
		$-(CH_3)_2$	1.21 (d)
**26**	Isokurkumenol*	=CH-	5.59 (br.s)
**27**	γ-Muurolene*	$-CH-C\underline{H}=$	5.50 (d)
		$=CH_2$	4.58 (m)
		$(CH_3)_2$	0.78 (d) and 0.90 (d)
**28**	1,1'-Oxydi-2-propanol*	$2x-CH_2-$	3.32 (dd) and 3.46 (dd)
		2xCH	3.95 – 4.05 (m)
		$2x\ CH_3$	1.15 (d)
**29**	α-Cedrene*	$CH-C\underline{H_3}$	0.84 (d)
		$-C\underline{H}-CH=$	2.17 (m)
		$-CH-C\underline{H}=$	5.22 (br.s)
**30**	Thujopsene*	$=C\underline{H}-CH_2-$	4.99 (m)

LP	Compound	Functional group	Chemical shift [ppm] (multiplet)
		$=CH-C\underline{H}_2-$	1.40 (m) and 1.78 (m)
		$=C-CH_3$	1.80 (br.s)
**31**	2,6-di-*tert*-butyl-4-methylphenol*	$2xH_{Ar}$	7.13 (s)
		-OH	5.14 (s)
		$-CH_3$	2.42 (s)
**32**	Cedrol*	$C\underline{H}_3-C-OH$	1.32 (br.s)
**33**	Methyl jasmonate*	$-C\underline{H}=CH-$	5.25 (m)
		-CH=CH-	5.46 (m)
		$-OCH_3$	3.69 (s)
**34**	Pyrrolo[3,2,1-*jk*]carbazole*	=CH	6.91 (d)
**35**	2-Methylbenzofuran*	$2xH_{Ar}$	7.26 (m)
		=CH	6.41 (td)
		$-CH_3$	2.50 (d)
**36**	(Z)-Ocimenone*	$-CH_3$	2.00 (d)
		$=CH_2$	5.53 (dd) and 5.64 (dd)
		=CH	6.17 (dq)
		=CH	6.40 (dd)
**37**	Ambrox*	$-CH_3$	1.08 (s)
**38**	Ketone musk*	$(CH_3)_3$	1.50 (s)
		$C(O)-CH_3$	2.45 (s)
**39**	2-ethylhexyl trans-4-methoxycinnamate	=CH	7.63 (d, J=16 Hz)
		$2xH_{Ar}$	7.47 (d)
		=CH	6.31 (d, J=16 Hz)
		$2xH_{Ar}$	6.88 (d)

LP	Compound	Functional group	Chemical shift [ppm] (multiplet)
		$-OCH_3$	3.71 (s)

*Compounds detected by means of GC/MS for original *Light Blue*, its substitutes and counterfeit version by Borowiecka *et al*.

4. PCA analysis conducted for full spectrum bucket tables.

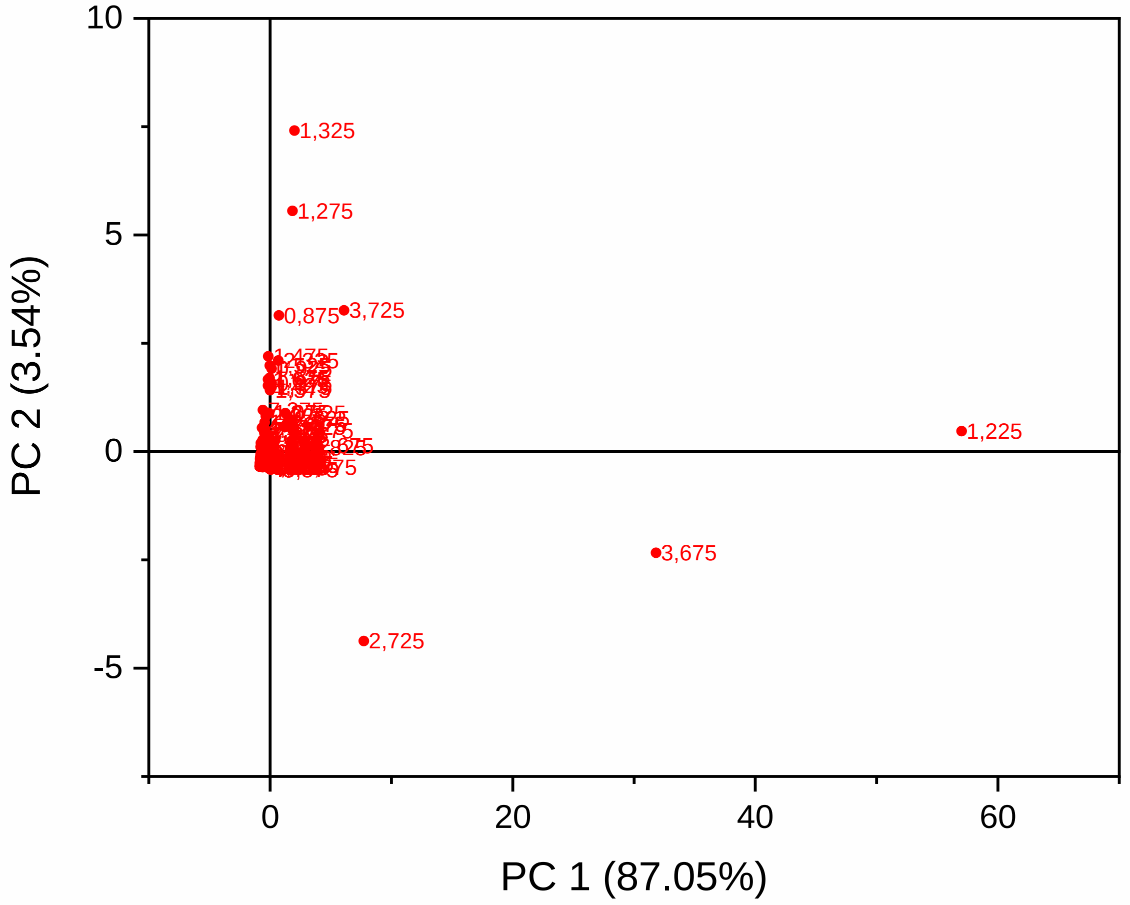

Figure S19 PCA loading plot generated from the ^{1}H NMR spectra from the authentic perfume and counterfeited and inspired samples for the range of chemical shifts from 0 to 10 ppm.

5. Comparison of the bucket tables from NMR spectra for two authentic samples.

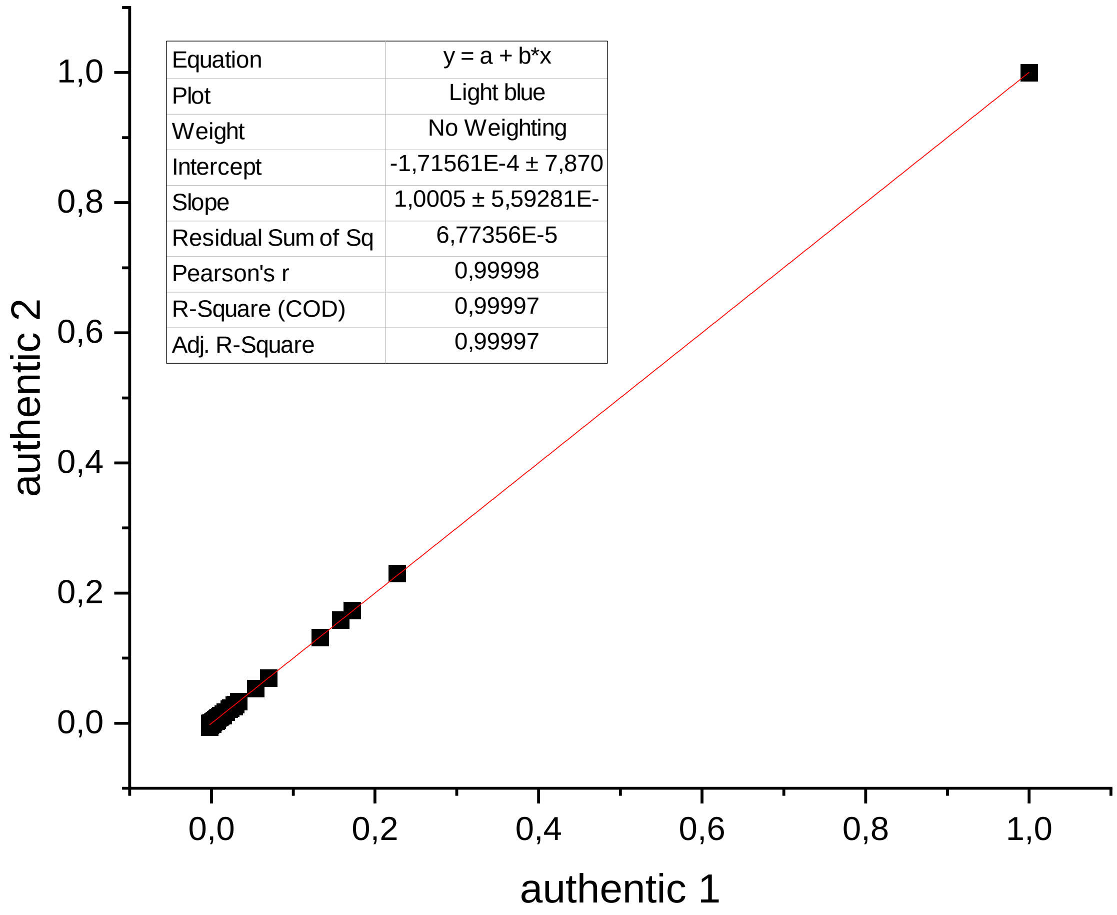

Figure S20 The comparison of the buckets from NMR spectra for two authentic samples of *Light blue*.

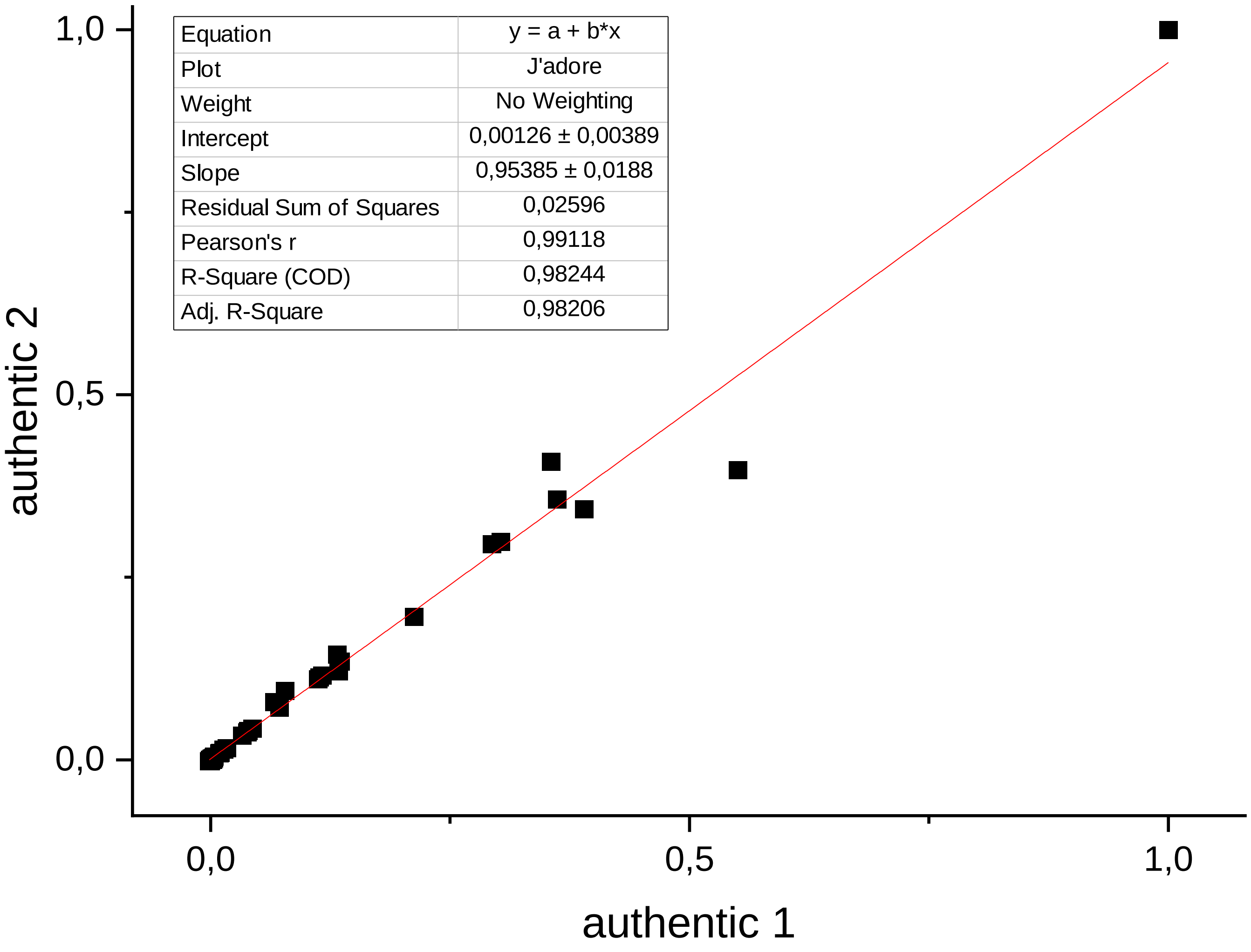

Figure S21 The comparison of the buckets from NMR spectra for two authentic samples of *J'adore*.
